# Multi-Action Planning for Threat Management: A Novel Approach for the Spatial Prioritization of Conservation Actions

**DOI:** 10.1371/journal.pone.0128027

**Published:** 2015-05-28

**Authors:** Lorenzo Cattarino, Virgilio Hermoso, Josie Carwardine, Mark J. Kennard, Simon Linke

**Affiliations:** 1 Australian Rivers Institute, Griffith University, Nathan, Queensland, Australia; 2 National Environmental Research Program Northern Australia Hub, Griffith University, Nathan, Queensland, Australia; 3 CSIRO Ecosystem Sciences, Dutton Park, Queensland, Australia; Fondazione Edmund Mach, Research and Innovation Centre, ITALY

## Abstract

Planning for the remediation of multiple threats is crucial to ensure the long term persistence of biodiversity. Limited conservation budgets require prioritizing which management actions to implement and where. Systematic conservation planning traditionally assumes that all the threats in priority sites are abated (*fixed prioritization approach*). However, abating only the threats affecting the species of conservation concerns may be more cost-effective. This requires prioritizing individual actions independently within the same site (*independent prioritization approach*), which has received limited attention so far. We developed an action prioritization algorithm that prioritizes multiple alternative actions within the same site. We used simulated annealing to find the combination of actions that remediate threats to species at the minimum cost. Our algorithm also accounts for the importance of selecting actions in sites connected through the river network (i.e., connectivity). We applied our algorithm to prioritize actions to address threats to freshwater fish species in the Mitchell River catchment, northern Australia. We compared how the efficiency of the independent and fixed prioritization approach varied as the importance of connectivity increased. Our independent prioritization approach delivered more efficient solutions than the fixed prioritization approach, particularly when the importance of achieving connectivity was high. By spatially prioritizing the specific actions necessary to remediate the threats affecting the target species, our approach can aid cost-effective habitat restoration and land-use planning. It is also particularly suited to solving resource allocation problems, where consideration of spatial design is important, such as prioritizing conservation efforts for highly mobile species, species facing climate change-driven range shifts, or minimizing the risk of threats spreading across different realms.

## Introduction

Long term persistence of many species is affected by a variety of different threatening processes which are heterogeneously distributed and require different conservation management actions. As conservation budgets are limited, it is important to invest resources in a cost-effective way. This requires identifying which conservation management actions (hereafter, *action*) should be prescribed for implementation, and where. Prioritizing more than one action simultaneously at the same site is critical to ensure long-term persistence of biodiversity, because most species are affected by more than one threat at the time, in the same site [1]. Although cost-effective prioritization of actions has received considerable attention in the literature [2–5], previous approaches have a limited capacity of prioritizing multiple actions within the same site.

Identifying priority sites and actions that aid preserving biodiversity in a cost-effective way is the aim of systematic conservation planning [6]. While systematic conservation planning has traditionally focused on prioritizing sites for conservation, there has been increasing attention on identifying which actions should be implemented where, in order to achieve conservation targets [7–9]. A key tenet of systematic conservation planning is to ensure that actions are prioritized based on how much new actions “complement” the benefit provided by the actions already selected [10]. This concept, known as complementarity, is essential to ensure that all species receive equal protection and to avoid duplication of conservation efforts [11]. Although traditionally applied in the context of selecting sites for reservation, the principle of complementarity has rarely been applied for prioritizing multiple actions [12]. Several previous multi-action prioritization approaches either did not account for complementarity [3, 13], or took complementarity in account but are not primarily spatial [5, 14]. Other studies, although had multiple actions available for selection at each site, only allowed one action per site to be prescribed [15–17]. Prioritizing more than one action within the same site, while accounting for complementarity, has received little attention.

Multiple actions within the same site should be prioritized independently (i.e., the benefits and costs of each action need to be assessed in isolation from the benefits and costs of other actions). By prioritizing different actions independently, we could reduce the costs of conservation, as only the actions needed to address the threats to the target species would be selected. Traditional tools to carry out spatial conservation prioritization, such as Marxan [18] and Zonation [19], assume that prioritizing sites for conservation is analogous to implementing the broader suit of actions necessary to protect biodiversity within the priority sites [4]. For example, Moilanen et al. [20] used the software Zonation to prioritize actions (e.g., fencing, riparian revegetation, construction of artificial wetlands) as fixed sets, which were either selected or not. Also, more recent software, such as Marxan with Zones [8], have been used to prescribe fixed sets of actions within priority sites [21–23]. These approaches assume that all the threats within a site need to be remediated and could not assess the benefits and costs of abating individual threats independently. Therefore, current complementarity-based approaches have a limited capacity of prioritizing independently more than one action within the same site.

The aim of this study is to develop a method for the spatial prioritization of actions to address threats to species. We developed a complementarity-based, optimization algorithm that prioritizes independently multiple actions within the same site and allowed more than one action per site to be prescribed. We also accounted for connectivity between the sites where actions are prioritized, which is critical to minimize the risk of threats propagating among priority sites [24]. We applied our approach to prioritize actions to address threats to freshwater fish species in the Mitchell River catchment, northern Australia. Freshwater biodiversity in the Mitchell River catchment is affected by a variety of threats, which are heterogeneously distributed and require different management actions [25]. We compared how the efficiency of our approach and the more traditional one, where all the threats within the priority sites are abated, varied, as the importance of achieving connectivity increased. We showed that our approach to threat management is more cost-effective than the traditional approach, particularly when the importance of achieving connectivity is high. Our novel methodological approach can aid decision makers to deliver cost-effective solutions for habitat restoration and land-use planning.

## Materials and Methods

### Multi-action allocation problem

We defined a multi-action allocation problem [26] where the aim was to find the set of actions that achieves the conservation target at the minimum cost. The target is the number of sites where each species had a particular benefit value. We interpreted the benefit for a species at a site as the presence (occurrence) of the species at the site, after actions have taken place and threats have been abated. In each site there were a number of threats and species. Different threats affected different species and each threat could affect more than one species. We used information on the ecological traits of species to define species-specific responses to threats (see below). We assumed that each threat could be abated by selecting one specific action and that when an action was selected the threat was completely abated and the species received full benefit [13]. We assumed this binomial response because our aim was to demonstrate the approach rather than estimating the exact benefits of conservation actions, which is a complex task [27], beyond the scope of this paper.

Species vulnerable to more than one threat might only benefit partially from abatement of a subset of the threats that affect them [13, 28]. Therefore, we assumed that the benefit for each species, at each site, depended on the number of threats, which affected the species at the site, and which had been abated, divided by the total number of threats affecting the species at the site. The benefit, *B*_*ji*_, of species *j* at site *i*, was expressed as follows:

$$B_{ji}={\left(\frac{\sum_{k=1}^{p}x_{ki}a_{ji}d_{ki}b_{jk}}{K_{ji}}\right)}^{3}$$ (1)

where *x*_*ki*_ is a control variable which equals 1 when threat *k* in site *i* is abated and 0 when threat *k* in site *i* is not abated; *a*_*ji*_ is a constant variable with a value of 1 when species *j* occurs in site *i* and 0 when species *j* does not occur in site *i*; *d*_*ki*_ is a constant variable with a value of 1 when threat *k* occurs in site *i* and 0 when threat *k* does not occur in site *i*; *b*_*jk*_ is a constant variable with a value of 1 when species *j* is vulnerable to threat *k* and 0 when species *j* is not vulnerable to threat *k*; and *K*_*ji*_ is the total number of threats that affect species *j* at site *i*. The value of *B*_*ji*_ increases slowly as the number of threats, which occur at site *i* and affect species *j*, and which are abated, increases, and it is maximum (i.e., 1) when all the threats to the species are abated (S1 Fig). This helped to avoid that only a subset of the threats affecting a species at a site was abated.

In order to ensure the achievement of targets, we assigned a penalty which was a function of the amount of target that had not been met, for each species. The cumulative species penalty, *Sp*, for all species was calculated as follows:

$$Sp=\sum_{j=1}^{n}SPF_{j}H(s_{j})s_{j}$$ (2)

where *n* is the number of species and *SPF* (Species Penalty Factor) is a scaling factor which determines the relative importance of meeting the target for each species. The Specie Penalty Factor was set to 10, which was the minimum value to ensure all targets were 100% met [29]. The Heaviside function, *H(s*_*j*_ *)*, is a step function which takes a value of zero when *s*_*j*_ ≤ 0 and 1 otherwise. The shortfall *s*_*j*_ represents how much of the target for each species is not met and is equal to $t_{j}-\sum_{i=1}^{m}B_{ji}$, where *t*_*j*_ is the target for species *j* and *m* is the number of sites. By subtracting the sum of the benefits across all the sites from the target, for each species, the shortfall is a measure of species complementarity (i.e., how much the benefits of abating the selected threats at one site complement the benefits of abating those threats at the other sites, for each species). Our target is a benefit of 1 in a maximum 100 sites, for each species. The target for those species that occurred in a number of sites smaller than 100, was set equal to the total number of sites where those species occurred, thus allowing full coverage of rare species with restricted ranges.

The degree to which the sites, where actions are prescribed, are connected to each other, through movement of individuals, matter or energy (i.e., connectivity), critically influences the effectiveness of conservation planning [24]. For example, in freshwater systems, prioritizing actions in sites that are connected through the river network is important to reduce the risk of threat propagation (e.g., increased sediment load in upstream sites, due to erosion of the river banks caused by domestic or feral herbivores trampling riparian vegetation, may propagate to downstream sites, where herbivores have been eliminated) [30]. We accounted for the upstream-downstream connections of riverine systems by assuming a penalty for each pair of sites connected by a river branch. Following Hermoso et al. [31] we defined a *connection* as the presence of real boundaries—two adjacent sites—or “virtual” ones—two non-adjacent sites (e.g., a headwater and a mouth site). The value of the penalty decreases as the distance between two sites increases, as the risk of threat propagation is higher when two sites are close to each other, than when they are further away. If at least one of the threats occurring within one site is abated, and none of the threats occurring within the upstream site is abated, the penalty for that missed connection is considered. The cumulative connectivity penalty, *Cp*, for all the connections was calculated as follows:

$$Cp=\sum_{i1=1}^{m}\;\sum_{i2=1}^{m}H\left(\sum_{k=1}^{p}x_{k\;i\;1}\right)\;\left[1-H\left(\sum_{k=1}^{p}x_{k\;i\;2}\right)\right]\;cv_{i\;1,\;i\;2}$$ (3)

where $H\left(\sum_{k=1}^{p}x_{k\;i\;1}\right)$ and $H\left(\sum_{k=1}^{p}x_{k\;i2}\right)$ are two Heaviside functions which take a value of 1 when $\sum_{k=1}^{p}x_{k\;i\;1}\geq 1$ and $\sum_{k=1}^{p}x_{k\;i\;2}\geq 1$; they take a value of 0 when $\sum_{k=1}^{p}x_{k\;i\;1}=0$ and $\sum_{k=1}^{p}x_{k\;i\;2}=0$. The variable *cv*_*i1*, *i2*_ is the penalty for the connection between site *i*1 and site *i*2. It ranges from 0 to 1 and is calculated as the inverse of the distance between the two sites [31]. The value of *Cp* in Eq 3 decreases as threats are abated in those sites located upstream of the sites already in the solution. However, when the selected upstream sites are close to the ones already in the solutions, *Cp* decreases more than when the selected upstream sites are further away from the sites already in the solutions.

The objective of our problem was to minimize the sum of the costs of selected actions and the connectivity penalties, subject to achieving the target for each species. Mathematically, our problem formulation is:

$$\text{min}\;\sum_{i=1}^{m}\sum_{k=1}^{p}c_{ik}x_{ki}+Sp+CSM\;Cp$$ (4)

$$\text{subject to}\;\sum_{i=1}^{m}\sum_{k=1}^{p}B_{ji}\geq t_{j}\;\forall j$$ (5)

where *c*_*ik*_ is the costs of the action required to abate threat *k* in site *i*, *x*_*ki*_ ∈ [0,1] is the abetment status of threat *k*, *Sp* is the species penalty (Eq 2), *CSM* (Connectivity Strength Modifier) is a scaling factor which controls the importance of minimizing the connectivity penalty in relation to the cost of the actions and the species target, and *Cp* is the connectivity penalty (Eq 3). When connectivity is not important in the optimization (*CSM* = 0), actions are selected in sites regardless of the location of those sites along the river network. As connectivity becomes more important (*CSM* > 0), actions tend to be selected in most of the sites upstream of the sites already in the solution.

### Action prioritization algorithm

**Fig 1:**
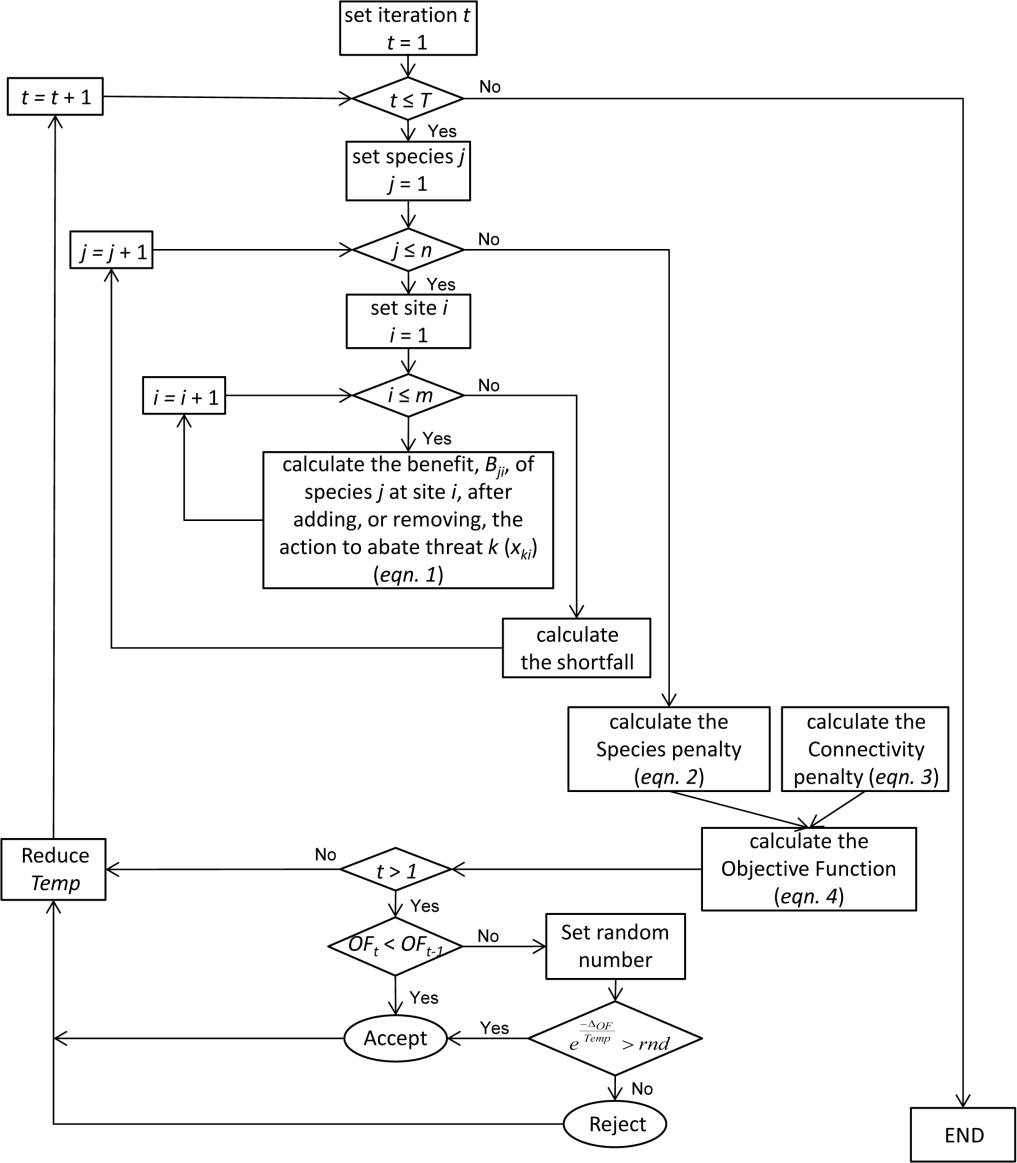
Schematic diagram of the steps of the action optimization algorithm. *T* is the number of iterations; *n* is the number of species; *m* is the number of sites; *Δ*_*OF*_ is the difference between the value of the objective function at iteration *t* and the value of the objective function at iteration *t*-1; *rnd* is a number randomly drawn from a uniform distribution; *Temp* is the temperature parameter.

We developed an action prioritization algorithm, which uses simulated annealing [32] to approximate the minimum value of the objective function (Eq 4). The algorithm iteratively adds or removes one action in one site, by changing the value of the control variable *x*_*ki*_, which represents the selection of the action required to abate threat *k* at site *i* (Fig 1). A complete description of the algorithm is reported in S1 Appendix. The input files required to run the algorithm are described in S2 Appendix. The action prioritization algorithm was implemented in the R programming language for statistical computing (version 3.1.0) [33].

### Case study: Mitchell River catchment

**Fig 2:**
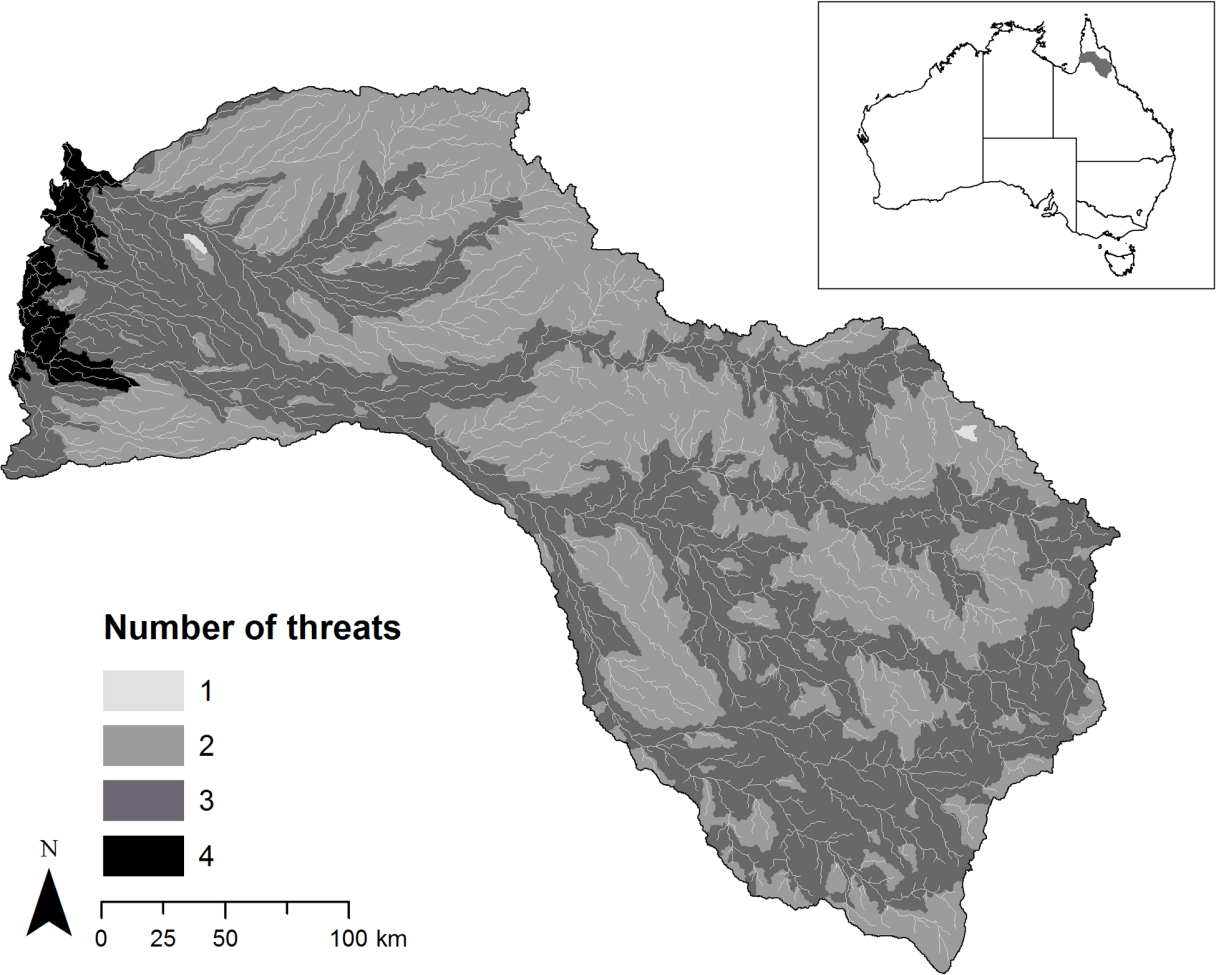
Spatial distribution of the occurrence of the threats in the case study area. Occurrence data represent presence or absence of a threat in each site. Threats include (1) water buffalo, (2) cane toad, (3) river flow alteration and (4) grazing land use.

We applied the action prioritization algorithm to finding the minimum set of actions to address threats to freshwater fish species in the Mitchell River catchment (northern Australia). Using ARC Hydro for AcrGIS 9.3 [34], we divided the whole catchment (71,630 km^2^) into 2,316 sites (i.e., sub-catchments), each one included the portion of river length between two consecutive river connections. We considered four major threats to freshwater fish species in the catchment: water buffalo (*Bubalis bubalis*), cane toad (*Bufo marinus*), river flow alteration (caused by impoundments, channels for water extractions and levee banks) and grazing land use [25]. We quantified the magnitude of each threat in each site by using estimates of the abundance of buffalos and toads [35], measures of flow regime alterations (Flow Regime Disturbance Index, FRDI) [36], and the proportion of grazing land use [37]. We characterized the occurrence (presence/absence) of each threat in the study area (Fig 2) by calculating whether in each site the magnitude of each threat was greater than (1), or equal to (0), zero.

We used the modelled spatial distribution of 44 fish species in the Mitchell river catchment [29, 38] as our conservation features. We defined the response of each species to each threat based on prior studies [39] and data on species-specific traits that may increase species vulnerability to threats [40, 41]. For instance, we assumed that presence of buffalos affected wetland-dependent species, which inhabit aquatic vegetation in the shallow margins of waterholes trampled by the buffalos. We also assumed that presence of cane toads affected predatory species, which may feed on the toxic toad larvae. We further assumed that altered flow regimes affect migratory species, whose movement may be impaired, particularly during the dry season, by the presence of road crossings and river gauging weirs. Finally, we assumed that grazing land use equally affected all the species considered, through increased sedimentation, nutrient enrichment and contamination with chemicals.

One action was available for remediating each specific threat: shooting for buffalo control, chemical or biological treatment for cane toad control, removal of dams or redesign of levee banks for flow-regime restoration and stewardship programs and pasture fencing for grazing management. We gave all actions the same unitary cost. However, we wanted to ensure that the cost of protecting a species in a site where no specific threat (i.e., buffalos, cane toads, river flow alteration and grazing land use) occurred was cheaper than the cost of protecting a species in a site where there were specific threats to be abated. Therefore, we considered a fifth action, “Land”, which represented the act of land acquisition, and which was needed in all sites and benefited all the species. In this way, the costs of protecting a species in a site where no threat occurred (i.e., the cost of land acquisition) was lower than the costs of protecting a species in a site where even one threat only occurred (i.e., cost of land acquisition + cost of the action targeting the specific threat). The cost of land acquisition reflected the administrative or transaction costs required for undertaking any type of action [42]. We also assumed that the cost of an action was constant across different sites (i.e., the cost of implementing an action in a site with low threat intensity, e.g., 10% of grazing land use, was the same as the cost of implementing an action in a site with high threat intensity, e.g., 100% of grazing land use).

### Analysis

To identify the minimum set of actions required to address the threats to freshwater fishes in the Mitchell River catchment, we ran the action prioritization algorithm 100 times (10^6^ iterations each). We considered different values of the *CSM* (0, 0.2, 0.3, 0.35, 0.4 and 0.7) to assess the effect of increasing the importance of achieving connectivity on the problem solution. We selected *CSM* values by trading off increasing importance of connectivity and minimizing the total area that needed to be protected [31]. We selected the run with the lowest objective function (best solution), for each value of *CSM*, and also recorded in which site which action was selected, the proportion of sites where each action was selected, out of the total number sites where the action was available for selection, and the total cost of the solution. We also calculated the number of times each action was selected across the total number of replicates (selection frequency). As a measure of performance, we calculated the efficiency of the solution as $1-\left(\frac{x}{t}\right)$, where *x* is the total cost of the selected actions and *t* is the sum of the costs of all available actions [43]. Efficiency decreases as the total cost of the selected actions increases.

We compared the performance of our approach, where multiple alternative threats can be abated independently in each site (*independent prioritization approach*), against the more traditional approach, where all the threats in a selected site are abated (*fixed prioritization approach*). The first approach was implemented using the action prioritization algorithm we developed, while for the second approach we used Marxan [18]. Marxan uses simulated annealing to minimize the value of an objective function, by varying the status (selected/not selected) of one site at the time. We run Marxan using exactly the same simulation framework we used when running the action optimization algorithm (conservation targets, no. of replicates, annealing parameters and outputs recorded). However, when running Marxan, we assumed that selection of a site corresponded to selection of the actions necessary to abate all the threats within the site. Therefore, the cost of each site was equal to the sum of the costs of the actions necessary to abate all the threats. In Marxan, the importance of achieving connectivity is controlled by the Boundary Length Modifier (*BLM*). We selected values of the *BLM* (0, 0.6, 0.67, 0.68, 0.69 and 0.7) so that the degree of connectivity achieved, for each value of *BLM* in Marxan, was similar to the degree of connectivity achieved, for each value of *CSM* in the action prioritization algorithm. The degree of connectivity was calculated as $1-\left(\frac{Cp}{Cp_{\max}}\right)$, where *Cp* is the connectivity penalty in the best solution and *Cp*_*max*_ is the highest connectivity penalty among all the best solutions, for each approach. The degree of connectivity increases as the connectivity penalty decreases.

## Results

**Fig 3:**
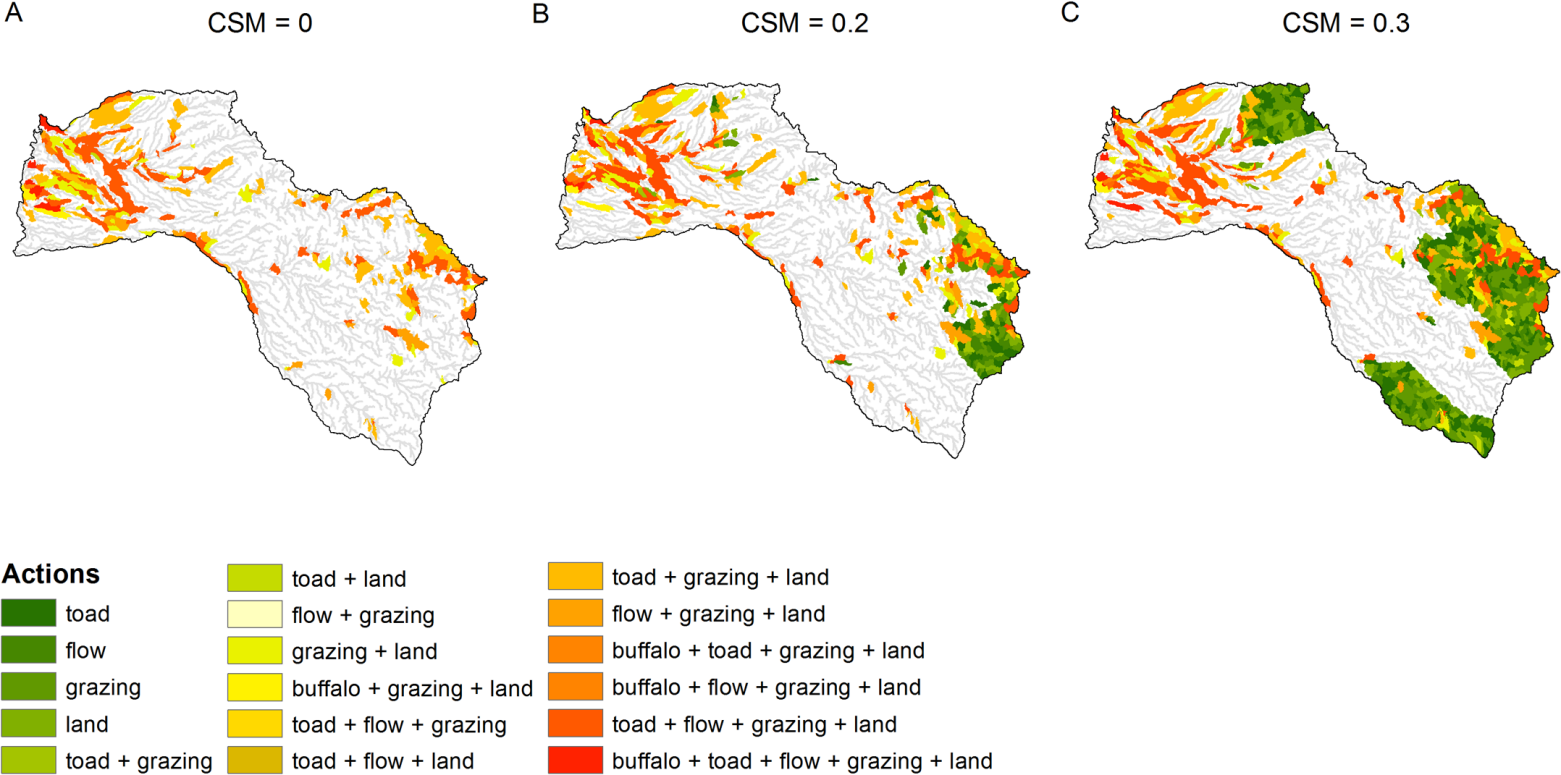
Spatial distribution of the actions selected by the action prioritization algorithm. Results are shown for increasing values of the Connectivity Strength Modifier (*CSM*).

When connectivity was not important (*CSM* = 0), the action prioritization algorithm selected grazing management, cane toad control, river flow restoration and land acquisition as priority actions, in most of the sites (Fig 3A). Buffalo control was also selected, particularly in the north-west part of the catchment (i.e., lowland areas). As the importance of connectivity increased (*CSM* = 0.2 and 0.3), one action (cane toad control, flow restoration, grazing management or land acquisition) was selected in most of the sites upstream of the ones already selected (Fig 3B–3C). The action prioritization algorithm converged well at the end of the annealing (S1–S6 Figs) and the actions identified as priority in the best solutions had also a quite high selection frequency, indicating that most of the replicate runs had similar solutions (S7–S12 Figs).

**Fig 4:**
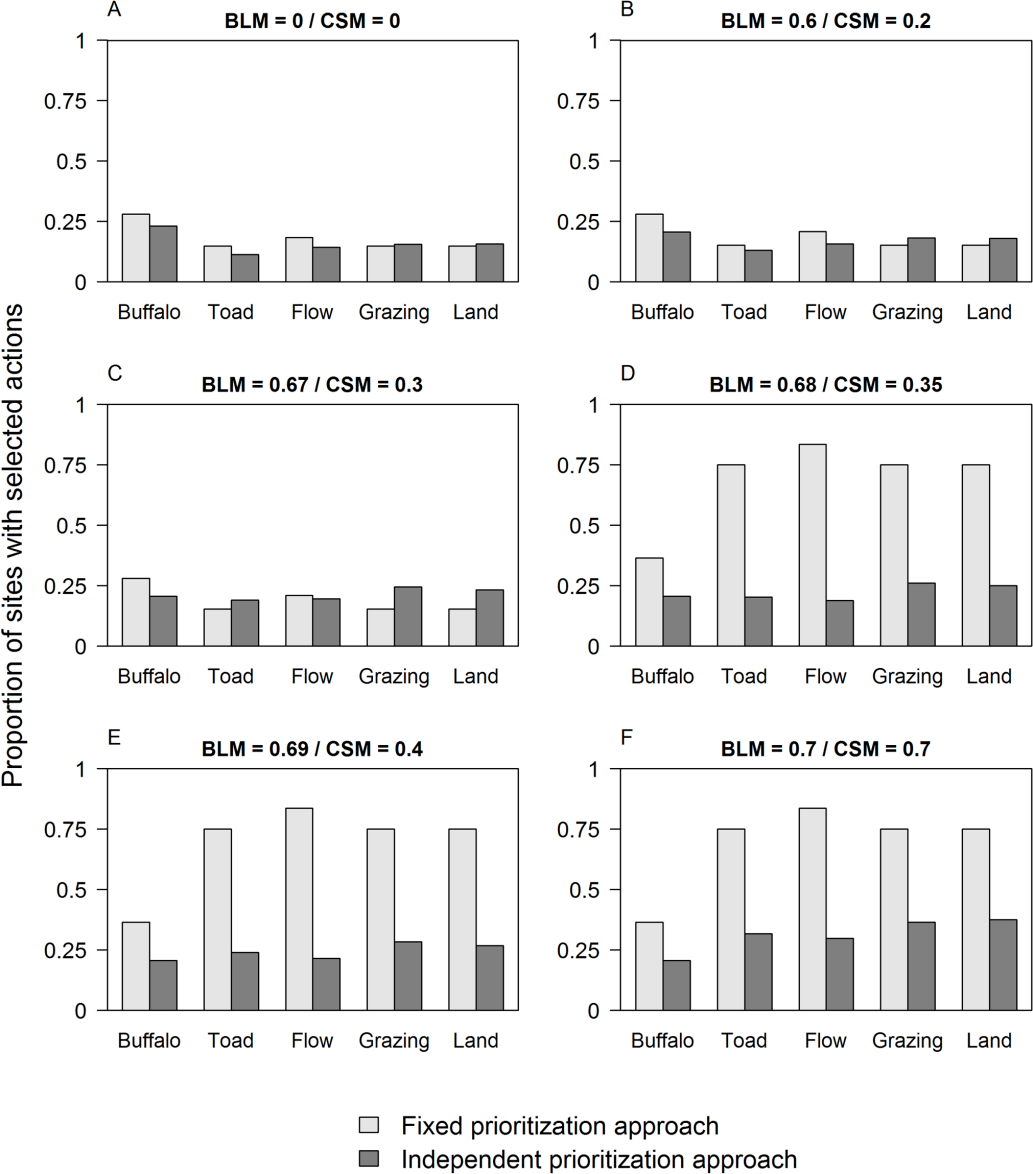
Proportion of sites where each action was selected for the fixed and the independent prioritization approach. The value on the y axis is calculated as number of sites where the action was selected out of the total number of sites where the action was available for selection. Results are shown for different values of *BLM* (Fixed Prioritization Approach) and *CSM* (Independent Prioritization Approach) (A—F).

When connectivity had no, or very low, importance, the proportion of sites where each action was selected was similar for the independent prioritization approach and the fixed prioritization approach (Fig 4A–4C). As the importance of connectivity increased, the independent prioritization approach selected actions in a lower proportion of sites than the fixed prioritization approach (Fig 4D–4F).

**Fig 5:**
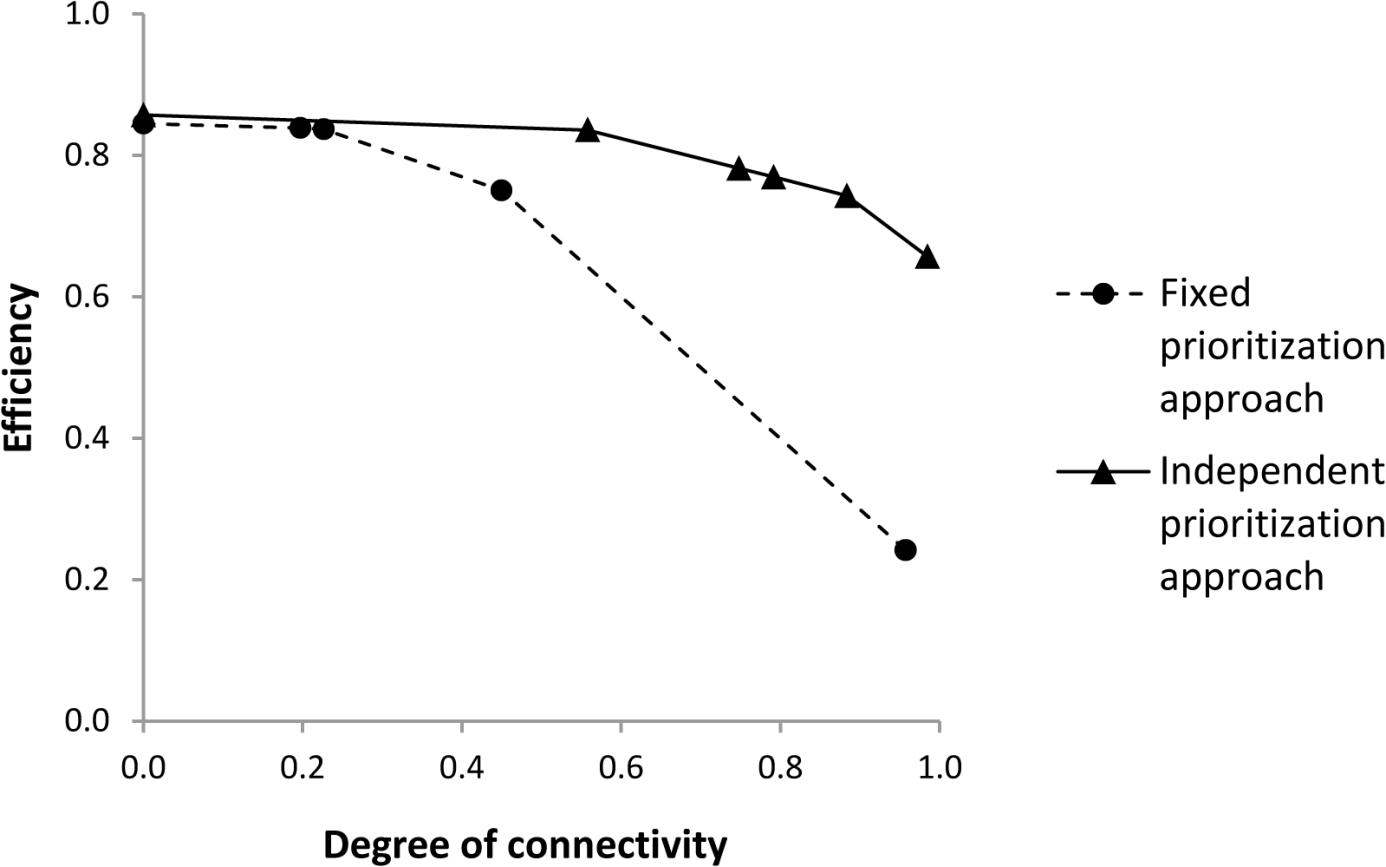
Efficiency of the best solution for increasing degrees of connectivity achieved in the best solution, and for different prioritization approaches. Efficiency is a measure of the ratio between the cost of the selected actions and the cost of all potential actions. The degree of connectivity is calculated as one minus the ratio of the connectivity penalty of the best solution, for a specific value of *BLM* (Fixed approach), or *CSM* (Independent approach), and the highest connectivity penalty among the best solutions for all values of *BLM*, or *CSM*. Dots represent the fixed prioritization approach, while triangles represent the independent prioritization approach.

When the degree of connectivity achieved was low (i.e., the importance of achieving connectivity was low), the efficiency of the independent prioritization approach was similar to the one of the fixed prioritization approach (Fig 5). As the degree of connectivity achieved increased (i.e., the importance of achieving connectivity increased), the efficiency of both approaches decreased. However, the efficiency of the fixed prioritization approach decreased faster than the efficiency of the individual prioritization approach (Fig 5). When the degree of connectivity achieved was close to one (i.e., the importance of achieving connectivity was very high), the individual prioritization approach delivered a solution with an efficiency almost 100% higher than the fixed prioritization approach.

## Discussion

Finding a cost-effective set of actions, and sites where to implement them, to improve biodiversity persistence, represents a key challenge in systematic conservation planning [7]. However, traditional conservation planning approaches are not well suited to prioritize multiple alternative actions within the same site. Here, we have addressed this gap by developing an algorithm that can prioritize multiple alternative actions within the same site, within a spatially-explicit framework that accounts for connectivity between priority sites. When connectivity is not important, our approach delivered planning solutions that are similar, in terms of efficiency, to the ones obtained with a more traditional approach, where all the threats, within a priority site, are abated. However, as the importance of connectivity increases, our approach outperforms the more traditional one. Our approach can be applied to solve cost-effectively complex ecological problems, where multiple threats affect multiple species in different ways, and the spatial configuration of priority sites is an important ecological consideration.

By identifying the specific actions necessary to remediate the threats affecting the target species in each site, our approach achieved higher efficiency than when prescribing remediation of all the threats in a selected site [20, 21]. This was true when the importance of achieving connectivity was even moderately low. The improvement that we demonstrate here is due to the fact that our approach selected each action in a lower number of sites compared to the traditional approach. By considering the direct relationships between the species and the threats occurring in each site, and the actions needed to remediate those threats, our approach can abate only those threats that specifically affect the species that require protection in each site, thus avoiding prescribing unnecessary actions. Our approach represents a major advance in systematic planning for threat management, as it allows to deliver solutions that are more costs-effective than the ones provided by current approaches.

Our approach represents an improvement over more recent spatial prioritization approaches. Levin et al [21] used Marxan with Zones to prioritize actions (e.g., fire management, revegetation, fencing) to achieve specific successional vegetation stages in a Mediterranean landscape. However, the authors prescribed a fixed set of actions in each priority site. Marxan with Zones prioritizes actions by allocating sites to spatial zones, where action are prescribed. Therefore, in order to prioritize independently multiple alternative actions within the same site, one zone needs to be created for each action, or combination of actions. In Marxan with Zones, connectivity can be accounted for by assigning to each pair of zones, a weight, which control the degree of spatial aggregation of sites between the zones. In our relatively simple problem with only 5 threats, and thus 2^5^ zones (32), the number of weight values to assign is $\frac{n!}{r!(n-r)!}$, where *n* is the total number of zones and *r* the number of elements in each pair of zones (2). This would mean parameterizing 496 values, which might be practically not possible. On the other hand, our approach allows both prioritizing independently actions within the same site and accounting for connectivity, without the need for zones and zone weights.

Previous attempts have achieved the simultaneous prioritization of multiple alternative actions, within the same site [15, 44]. However, to the best of our knowledge, one previous multi-action study allowed more than one action to be prescribed within the same priority site. Carwardine et al. [1] used Marxan to prioritize different actions across five sites, by defining pseudo sites that represented a unique combination of sites and actions. However, this approach was carried out at a coarse resolution (sites = bioregions) that will necessarily limit the spatial precision of where to implement actions. Furthermore, the pseudo-site approach might not be able to deal with the issue of river connectivity in a cost-effective way. When creating pseudo sites, all the connections between pseudo sites have the same connectivity penalty value of the connection between their normal sites, as pseudo sites are topologically equivalent to normal sites (they are in the same location as the normal site, relative to the other sites). Therefore, the algorithm would tend to select all the actions within a site (i.e., all the pseudo sites), to minimize the connectivity penalty. This would result in an overrepresentation of actions, which would reduce the efficiency of the solution.

Interestingly, when connectivity is not an important consideration in the optimization, the efficiency of the independent approach is similar to the efficiency of the fixed approach. This might be due to the spatial overlap of species that required different actions. When connectivity is not important, actions are selected in sites based only on their contribution towards species target. Therefore, some sites might always be selected, regardless of the approach used, simply because of the spatial co-occurrence of species. Let’s assume that species *X* and *Y* occur at site *i*, where there are three actions available for selection (A, B and C). If species *X* requires action A and B, and species *Y* requires action C, following our independent prioritization approach, protection of species *X* and *Y* at site *i*, requires prioritizing all the actions available at site *i*, which happens to be the same strategy followed by the fixed prioritization approach. As the importance of connectivity increases, the sites where actions are selected need to be located upstream of the sites already in the solution. In this case, while the fixed approach selects always all actions in the upstream sites, the independent approach can select only the actions needed to protect one species (and not all species) in the upstream site, as that is enough to reduce the connectivity penalty.

Although we have demonstrated the application of our approach to the freshwater realm, our approach can be applied to other realms as well. Prioritizing conservation actions in freshwater systems, while maintaining functional ecological processes, requires accounting for river connectivity [45]. This is a key challenge because planning units that are far apart from each other can still be connected by flow of materials and energy (“virtual” connections). This situation is similar in the marine realm where, for example, coral reefs are connected through major routes of larval dispersal [46]. By only considering “real” connections (two adjacent planning units), rather than “virtual” ones as well, our approach can be easily applied to the terrestrial realm [18]. Our framework is suited to solve a range of spatial conservation prioritization problems, where connectivity between priority sites is an important consideration. These include: accounting for shifts in the range of species affected by climate change into the prioritization of conservation actions [47], incorporating larval dispersal into the design of marine protected areas [48], and, more generally, achieving cost-effective planning across different environmental realms (terrestrial, freshwater or marine) [12].

We made three key assumptions which offer scope for future research. First, we assumed that an action was either implemented or not. In reality, however, actions can be implemented to different intensity levels, based on the intensity of each threat in each site [27]. Assuming that abating a threat at two different sites provides similar benefits for a species, it might be cheaper to focus on the site where the intensity of the threat is low (e.g., low buffalo abundance) than the site where the intensity of the threat is high (e.g., high buffalo abundance). Accounting for threat, and therefore action, intensity may further increase the efficiency of our approach and it is something that future research should consider exploring.

Second, we assumed that a species fully benefited from abatement of a threat. From that it followed that a species affected by more than one threat in a site received full benefit only when all the threats to the species were remediated, as we did not have information on which was the key threat affecting each species. As a result of this assumption, actions may have been selected in less threatened sites, where there is a small number of threats. However, conservation efforts can also be focused in more threatened sites, where the number of threats is high but the returns for biodiversity are also high [49]. Future applications of our approach include incorporating ecological responses of species to different threat intensities into the spatial prioritization framework [12].

Third, we assumed that two sites were connected when anyone of the threats occurring in each site was abated; hence, we only considered connectivity between the sites where threats were abated. However, to fully account for spatially-explicit processes between biodiversity features, connectivity between sites where the same threat was abated can be as important. A classic example comes from riverine settings, where the negative consequences of a threat occurring in an upstream site (e.g., weed eradication) might propagate to downstream areas, where the same threat has already been abated [50]. Accounting for connectivity between sites where the same threat is abated should be a key aspect to develop in future applications.

## Conclusions

We have developed a novel methodological approach for prioritizing multiple alternative actions, within a spatially-explicit framework that accounts for connectivity between the sites where actions are prescribed. Our approach is particularly suited to solving complex problems where conservation management needs to spatially allocate different actions, with different impacts on biodiversity. Examples include: prescribing, and scheduling, allocation of multiple habitat restoration actions within a site [51], and cost-effective land use planning to conserve species with contrasting habitat requirements [52]. Our approach represents a cost-effective and generalizable way for conducting spatially-explicit prioritization of actions for threat management.

## Supporting Information

S1 Appendix Description of the action prioritization algorithm. (DOCX)

S2 Appendix Input files required to run the action prioritization algorithm. (DOCX)

S1 Fig Species benefit as the number of threats abated increases. The graph shows the value of the species benefit at a site, as the number of threats, which occur at the site and affect the species, and which are abated, increases. Different lines represent different total numbers of threats that affects the species at the site.
(TIF)

S2 Fig Cost, Species penalty and Connectivity penalty, as the number of annealing iterations of the best solution increases, for *CSM* = 0. The values of Species and Connectivity penalty are weighted by their respective scaling factors (i.e., *SPF* and *CSM*). “Cost” is measured as number of actions selected; “Species penalty” as the number of sites where each species does not have a benefit of 1; and “Connectivity penalty” as the inverse of the squared distance (1/km^2^) between pairs of sites, where one of the sites is not in the solution.
(TIF)

S3 Fig Cost, Species penalty and Connectivity penalty, as the number of annealing iterations of the best solution increases, for *CSM* = 0.2. The values of Species and Connectivity penalty are weighted by their respective scaling factors (i.e., *SPF* and *CSM*). “Cost” is measured as number of actions selected; “Species penalty” as the number of sites where each species does not have a benefit of 1; and “Connectivity penalty” as the inverse of the squared distance (1/km^2^) between pairs of sites, where one of the sites is not in the solution.
(TIF)

S4 Fig Cost, Species penalty and Connectivity penalty, as the number of annealing iterations of the best solution increases, for *CSM* = 0.3. The values of Species and Connectivity penalty are weighted by their respective scaling factors (i.e., *SPF* and *CSM*). “Cost” is measured as number of actions selected; “Species penalty” as the number of sites where each species does not have a benefit of 1; and “Connectivity penalty” as the inverse of the squared distance (1/km^2^) between pairs of sites, where one of the sites is not in the solution.
(TIF)

S5 Fig Cost, Species penalty and Connectivity penalty, as the number of annealing iterations of the best solution increases, for *CSM* = 0.35. The values of Species and Connectivity penalty are weighted by their respective scaling factors (i.e., *SPF* and *CSM*). “Cost” is measured as number of actions selected; “Species penalty” as the number of sites where each species does not have a benefit of 1; and “Connectivity penalty” as the inverse of the squared distance (1/km^2^) between pairs of sites, where one of the sites is not in the solution.
(TIF)

S6 Fig Cost, Species penalty and Connectivity penalty, as the number of annealing iterations of the best solution increases, for *CSM* = 0.4. The values of Species and Connectivity penalty are weighted by their respective scaling factors (i.e., *SPF* and *CSM*). “Cost” is measured as number of actions selected; “Species penalty” as the number of sites where each species does not have a benefit of 1; and “Connectivity penalty” as the inverse of the squared distance (1/km^2^) between pairs of sites, where one of the sites is not in the solution.
(TIF)

S7 Fig Cost, Species penalty and Connectivity penalty, as the number of annealing iterations of the best solution increases, for *CSM* = 0.7. The values of Species and Connectivity penalty are weighted by their respective scaling factors (i.e., *SPF* and *CSM*). “Cost” is measured as number of actions selected; “Species penalty” as the number of sites where each species does not have a benefit of 1; and “Connectivity penalty” as the inverse of the squared distance (1/km^2^) between pairs of sites, where one of the sites is not in the solution.
(TIF)

S8 Fig Selection frequency of different actions for *CSM* = 0. Selection frequency is calculated as the number of times each action is selected across 100 replicates. Actions are: buffalo control (“Buffalo”), cane toad control (“Cane Toad”), river flow-regime restoration (“Flow”), grazing management (“Grazing”) and land acquisition (“Land”).
(TIF)

S9 Fig Selection frequency of different actions for *CSM* = 0.2. Selection frequency is calculated as the number of times each action is selected across 100 replicates. Actions are: buffalo control (“Buffalo”), cane toad control (“Cane Toad”), river flow-regime restoration (“Flow”), grazing management (“Grazing”) and land acquisition (“Land”).
(TIF)

S10 Fig Selection frequency of different actions for *CSM* = 0.3. Selection frequency is calculated as the number of times each action is selected across 100 replicates. Actions are: buffalo control (“Buffalo”), cane toad control (“Cane Toad”), river flow-regime restoration (“Flow”), grazing management (“Grazing”) and land acquisition (“Land”).
(TIF)

S11 Fig Selection frequency of different actions for *CSM* = 0.35. Selection frequency is calculated as the number of times each action is selected across 100 replicates. Actions are: buffalo control (“Buffalo”), cane toad control (“Cane Toad”), river flow-regime restoration (“Flow”), grazing management (“Grazing”) and land acquisition (“Land”).
(TIF)

S12 Fig Selection frequency of different actions for *CSM* = 0.7. Selection frequency is calculated as the number of times each action is selected across 100 replicates. Actions are: buffalo control (“Buffalo”), cane toad control (“Cane Toad”), river flow-regime restoration (“Flow”), grazing management (“Grazing”) and land acquisition (“Land”).
(TIF)

S13 Fig Selection frequency of different actions for *CSM* = 0.4. Selection frequency is calculated as the number of times each action is selected across 100 replicates. Actions are: buffalo control (“Buffalo”), cane toad control (“Cane Toad”), river flow-regime restoration (“Flow”), grazing management (“Grazing”) and land acquisition (“Land”).
(TIF)

S1 Table Values of the simulated annealing parameters used in the action prioritization algorithm. (XLSX)
