# Supplementary material for: Multi-Action Planning for Threat Management: A Novel Approach for the Spatial Prioritization of Conservation Actions
Source: PLoS One. 2015 May 28;10(5):e0128027. doi: 10.1371/journal.pone.0128027 (PMC4447389; doi:10.1371/journal.pone.0128027)
Supplement: S1 Appendix — (DOCX) [file pone.0128027.s001.docx]

Appendix S1. Description of the action prioritization algorithm

We used simulated annealing [[1](#_ENREF_1)] to minimize the value of the objective function by changing the value of the control variable *x_ki_*, which represents the selection of the action required to abate threat *k* at site *i*. The action prioritization algorithm starts with an initial proportion of actions, *P_i_*, selected randomly from the pool of all the available ones (Table S1). First, the algorithm calculates the benefit of the selected actions for each species at each site. The benefit for each species, at each site, depends on the number of threats, which affect the species at the site, and which have been abated, divided by the total number of threats affecting the species at the site. The benefit, *B_ji_*, of species *j* at site *i*, is expressed as follows:

|  | $B_{ji}=\left(\frac{\sum_{k=1}^{p} x_{ki}a_{ji}d_{ki}b_{jk}}{K_{ji}}\right)^{3}$ |  |
| --- | --- | --- |

where *x_ki_* is a control variable which equals 1 when threat *k* in site *i* is abated and 0 when threat *k* in site *i* is not abated; *a_ji_* is a constant variable with a value of 1 when species *j* occurs in site *i* and 0 when species *j* does not occur in site *i*; *d_ki_* is a constant variable with a value of 1 when threat *k* occurs in site *i* and 0 when threat *k* does not occur in site *i*; *b_jk_* is a constant variable with a value of 1 when species *j* is vulnerable to threat *k* and 0 when species *j* is not vulnerable to threat *k*; and *K_ji_* is the total number of threats that affect species *j* at site *i*. The value of *B_ji_* increases slowly as the number of threats, which occur at site *i* and affect species *j*, and which are abated, increases, and it is maximum (i.e., 1) when all the threats to the species are abated. This helps to avoid that only a subset of the threats affecting a species at a site is abated.

Second, the algorithm calculates the species penalty (*Sp*). The cumulative species penalty, *Sp*, for all species is calculated as follows:

|  | $Sp=\sum_{j=1}^{n} SPF_{j}H(s_{j})s_{j}$ |  |
| --- | --- | --- |

where *n* is the number of species and *SPF* (Species Penalty Factor) is a scaling factor which determines the relative importance of meeting the target for each species. The Heaviside function, *H(s_j_)*, is a step function which takes a value of zero when *s_j_* ≤ 0 and 1 otherwise. The shortfall *s_j_* represents how much of the target for each species is not met and is equal to $t_{j}-\sum_{i=1}^{m} B_{ji}$, where *t_j_* is the target for species *j* and *m* is the number of sites. By subtracting the sum of the benefits across all the sites from the target, for each species, the shortfall is a measure of species complementarity (i.e., how much the benefits of abating the selected threats at one site complement the benefits of abating those threats at the other sites, for each species). The target is a benefit of 1 in a maximum 100 sites, for each species. The target for those species that occur in a number of sites smaller than 100, is set equal to the total number of sites where those species occur, thus allowing full coverage of rare species with restricted ranges.

The algorithm then calculates the connectivity penalty (*Cp*). The cumulative connectivity penalty, *Cp*, for all the connections is calculated as follows:

|  | $Cp=\sum_{i 1=1}^{m} \sum_{i 2=1}^{m} H\left(\sum_{k=1}^{p} x_{k i 1}\right) \left[ 1-H\left(\sum_{k=1}^{p} x_{k i 2}\right) \right] cv_{i 1, i 2}$ |  |
| --- | --- | --- |

where $H\left(\sum_{k=1}^{p} x_{k i 1}\right)$ and $H\left(\sum_{k=1}^{p} x_{k i 2}\right)$ are two Heaviside functions which take a value of 1 when $\sum_{k=1}^{p} x_{k i 1}\geq1$and $\sum_{k=1}^{p} x_{k i 2}\geq1$; they take a value of 0 when $\sum_{k=1}^{p} x_{k i 1}=0$and $\sum_{k=1}^{p} x_{k i 2}=0$. The variable *cv_i1, i2_* is the penalty for the connection between site *i*1 and site *i*2. It ranges from 0 to 1 and is calculated as the inverse of the distance between the two sites [[2](#_ENREF_2)]. The value of *Cp* in eqn. 3 decreases as threats are abated in those sites located upstream of the sites already in the solution. However, when the selected upstream sites are close to the ones already in the solutions, *Cp* decreases more than when the selected upstream sites are further away from the sites already in the solutions.

Finally, the algorithm sums all the above components in the objective function. The objective of our problem is to minimize the sum of the costs of selected actions and the connectivity penalties, subject to achieving the target for each species. Mathematically, our problem formulation is:

|  | min $\sum_{i=1}^{m} \sum_{k=1}^{p} c_{ik}x_{ki}+Sp+CSM Cp$ |  |
| --- | --- | --- |
|  | subject to $\sum_{i=1}^{m} \sum_{k=1}^{p} B_{ji}\geq t_{j} \forall j$ |  |

where *c_ik_* is the costs of the action required to abate threat *k* in site *i*, $x_{ki}\in[0, 1]$ is the abetment status of threat *k*, *Sp* is the species penalty (eqn 2), *CSM* (Connectivity Strength Modifier) is a scaling factor which controls the importance of minimizing the connectivity penalty in relation to the cost of the actions and the species target, and *Cp* is the connectivity penalty (eqn 3). When connectivity is not important in the optimization (*CSM* = 0), actions are selected in sites regardless of the location of those sites along the river network. As connectivity becomes more important (*CSM* > 0), actions tend to be selected in most of the sites upstream of the sites already in the solution.

At each of the following iterations, one site/threat combination is sampled randomly from a uniform distribution of all the site/threat combinations. The action required to abate the threat at the site, is then selected, or deselected, if the action is already in the solution. The new value of the objective function after the change is calculated and the change accepted if the new value is smaller than the previous one (good change). Bad changes (i.e., the new value is larger than the previous one) are accepted with probability *Pr*, $e^{\frac{-\Delta_{OF}}{Temp}}$, where *Δ_OF_* is the difference between the value of the objective function at iteration *t* and the value of the objective function at iteration *t* -1, and *Temp* is the temperature parameter. A random number between 0 and 1 is drawn from a uniform distribution. If the random number is greater than *Pr*, the bad change is accepted, otherwise it is rejected. The initial temperature value is *Temp_0_*, which decreases, at each iteration, with rate *r* (cooling rate) (Table S1). Therefore, as the number of iterations increases, *Pr* decreases, and thus only good changes are accepted.

References

1. Kirkpatrick S, Gelatt CD, Vecchi MP (1983) Optimization by simulated annealing. Science 220: 671-680.

2. Hermoso V, Linke S, Prenda J, Possingham HP (2011) Addressing longitudinal connectivity in the systematic conservation planning of fresh waters. Freshwater Biology 56: 57-70.
