# Supplementary material for: Multi-Action Planning for Threat Management: A Novel Approach for the Spatial Prioritization of Conservation Actions
Source: PLoS One. 2015 May 28;10(5):e0128027. doi: 10.1371/journal.pone.0128027 (PMC4447389; doi:10.1371/journal.pone.0128027)
Supplement: S2 Appendix — (DOCX) [file pone.0128027.s002.docx]

Appendix S2. Input files required to run the action prioritization algorithm

Different input files are required to run the action prioritization algorithm (Table S2.1). Some of them, such as the spatial distribution of conservation features, i.e. species, (“Conservation Feature”), the amount of each conservation feature in each planning unit (“Planning Unit vs. Conservation Feature”) and the value of penalty for each missed connection (“Boundary Length”), are similar to the input files required to run other reserve design algorithm, such as Marxan [[1](#_ENREF_1)] and Marxan with Zones [[2](#_ENREF_2)]. In addition, the action prioritization algorithm requires information on the cost of actions in each planning unit, the spatial distribution of threats, the relationship between threats and conservation features, and the relationship between actions and threats. All input files use the .csv extension.

| **Table S2.1. Input files required to run the action prioritization algorithm.** | | |
| --- | --- | --- |
| **Input file** | **Description** | **Structure** |
| Conservation Feature file | Identifier (id), target and Species Penalty Factor (*SPF*) of each conservation feature | Matrix with id, target and SPF (columns) of each conservation feature (rows) (Fig. S2.1) |
| Site vs. Action file | Cost of each action in each site | Matrix with the cost of each action (columns) in each site (rows) (Fig. S2.2) |
| Site vs. Conservation Feature file | Amount of each conservation feature in each site | Matrix with the amount of each conservation feature (columns) in each site (rows) (Fig. S2.3) |
| Boundary file | Site 1 and 2 ids, number of links (i.e., portions of river length between the two sites) and penalty, for each pair of sites | Matrix with site 1 id, site 2 id, number of links and penalty (columns), for each pair of sites (rows) (Fig. S2.4) |
| Site vs. Threat file | Occurrence (presence/absence) of each threat in each site | Matrix with occurrence of each threat (columns) in each site (rows) (Fig. S2.5) |
| Conservation Feature vs. Threat file | Relationship indicating whether a conservation feature is affected by each threat | Matrix with a value of 1 when the conservation feature is affected by the threat, and a 0 when the conservation feature is not affected by the threat, for each threat (columns) and conservation feature (rows) (Fig. S2.6) |
| Action vs. Threat file | Relationship indicating whether an action remediates a threat | Matrix with a value of 1 when the action remediates the threat, and a 0 when the action does not remediate the threat, for each action (columns) and threat (rows) (Fig. S2.7) |


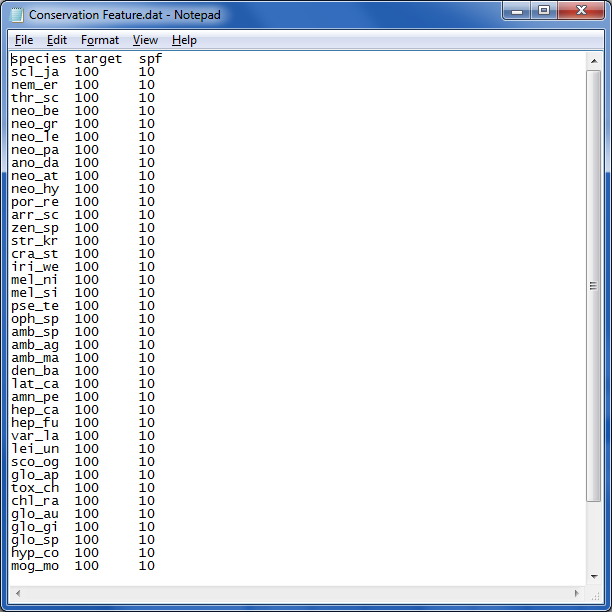


Fig. S2.1. Example of the Conservation Feature file used in the action optimization algorithm.


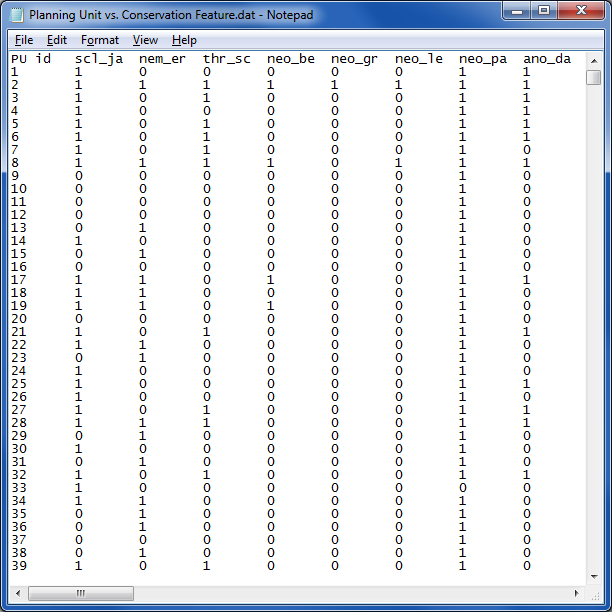


Fig. S2.2. Example of the Planning Unit vs. Conservation Feature file used in the action optimization algorithm.


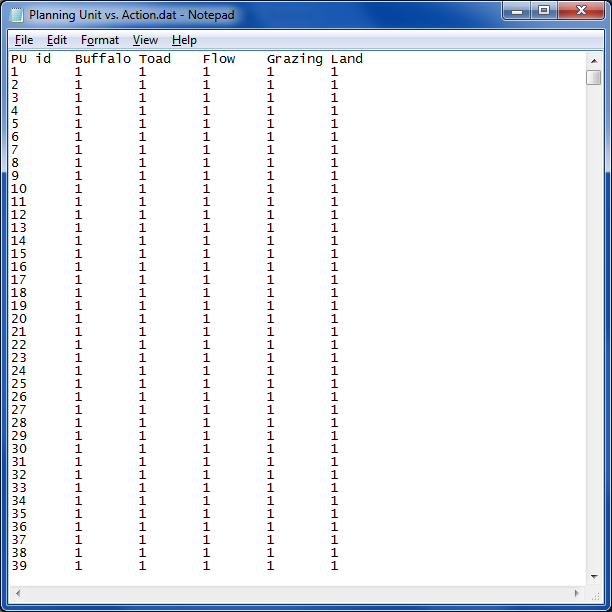


Fig. S2.3. Example of the Planning Unit vs. Action file used in the action optimization algorithm.


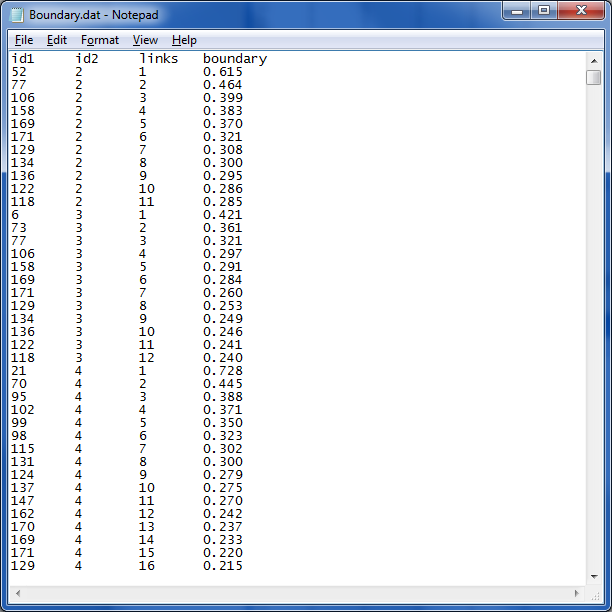


Fig. S2.4. Example of the Boundary file used in the action optimization algorithm.


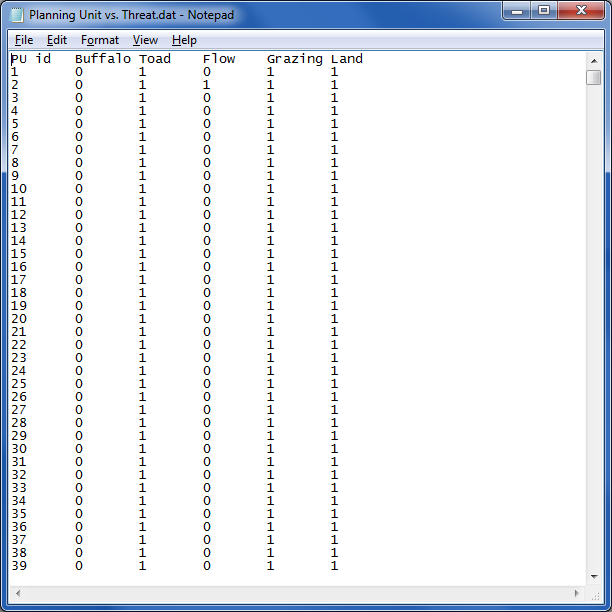


Fig. S2.5. Example of the Planning Unit vs. Threat file used in the action optimization algorithm.


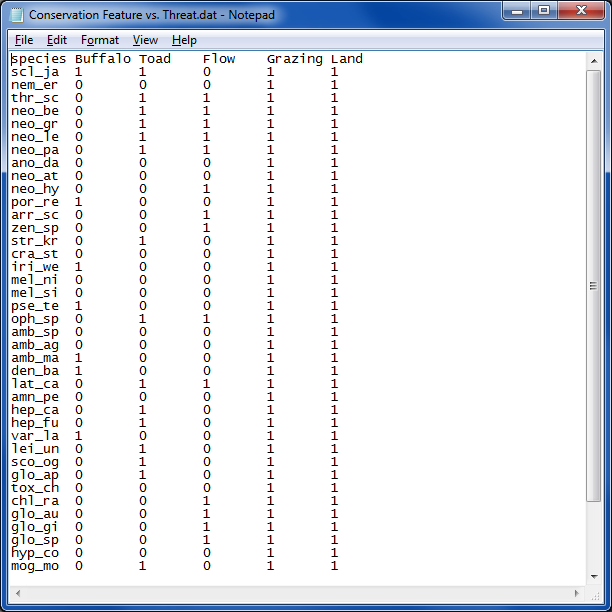


Fig. S2.6. Example of the Conservation Feature vs. Threat file used in the action optimization algorithm.


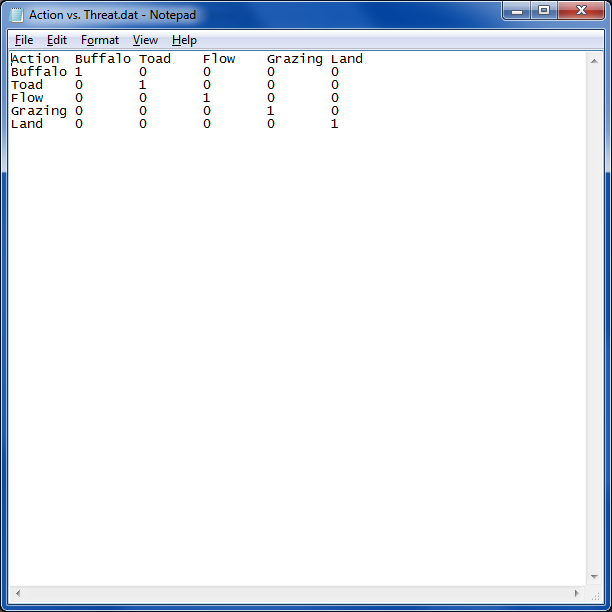


Fig. S2.7. Example of the Action vs. Threat file used in the action optimization algorithm.

References

1. Ball IR, Possingham HP, Watts ME (2009) Marxan and relatives: softwares for spatial conservation prioritization. In: Moilanen A, Wilson KA, Possingham HP, editors. Spatial Conservation Prioritization: quantitative methods and computational tools. Oxford, UK: Oxford University Press. pp. 185-195.

2. Watts ME, Ball IR, Stewart RS, Klein CJ, Wilson K, et al. (2009) Marxan with Zones: Software for optimal conservation based land- and sea-use zoning. Environmental Modelling & Software 24: 1513-1521.
