# Supplementary figures and images for: Multi-Action Planning for Threat Management: A Novel Approach for the Spatial Prioritization of Conservation Actions
Source: PLoS One. 2015 May 28;10(5):e0128027. doi: 10.1371/journal.pone.0128027 (PMC4447389; doi:10.1371/journal.pone.0128027)

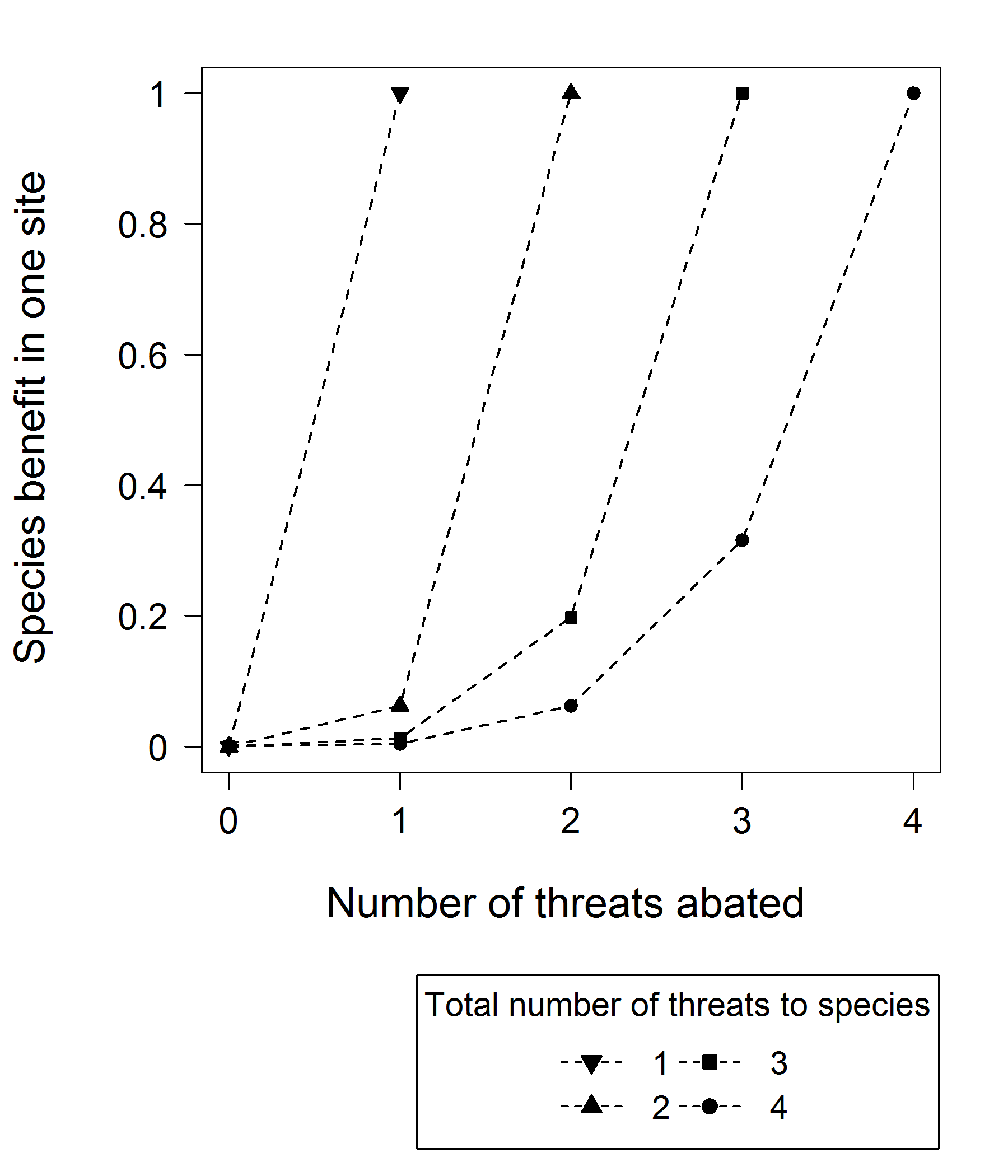

Supplement: S1 Fig — The graph shows the value of the species benefit at a site, as the number of threats, which occur at the site and affect the species, and which are abated, increases. Different lines represent different total numbers of threats that affects the species at the site. (TIF) [file pone.0128027.s003.tif]

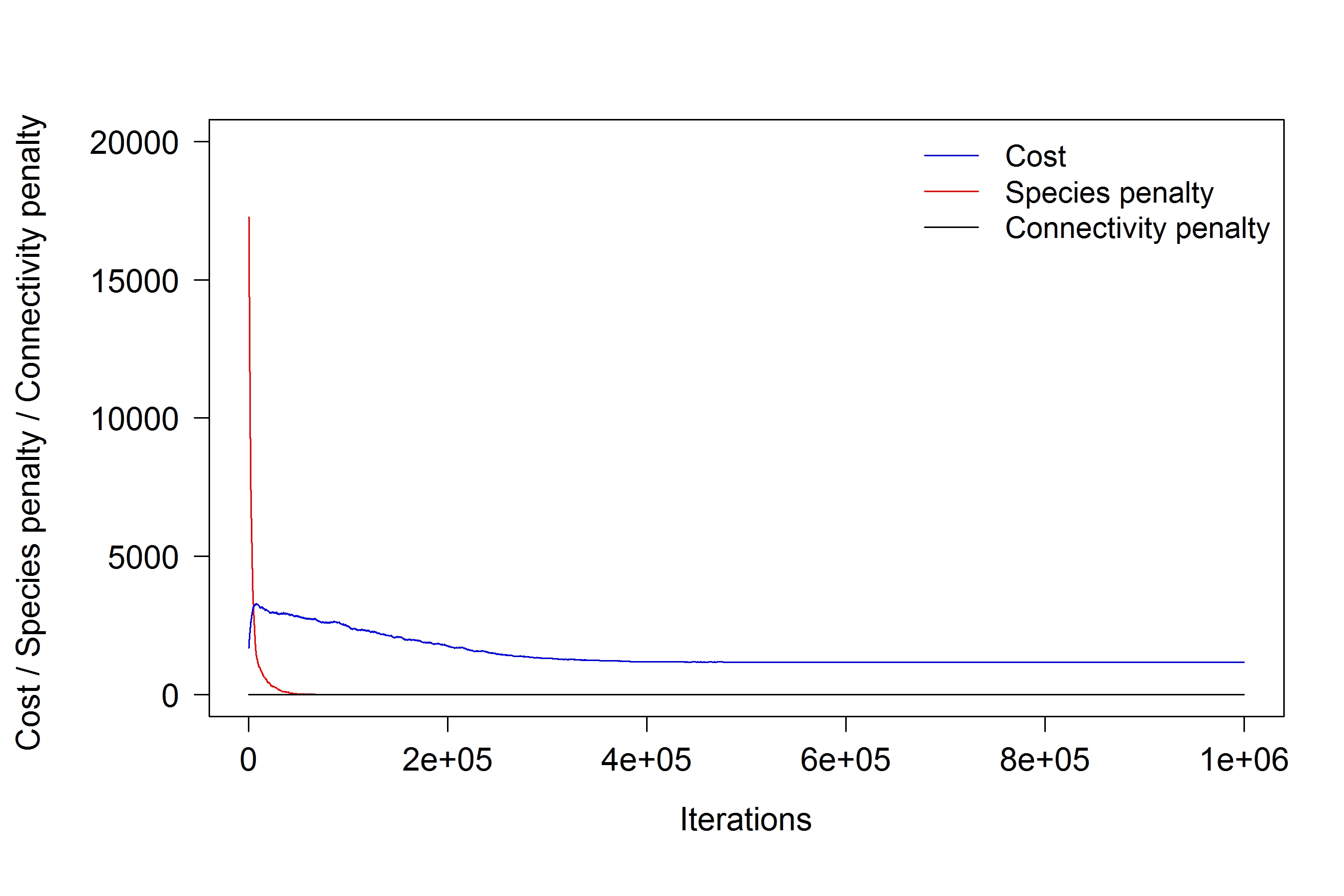

Supplement: S2 Fig — The values of Species and Connectivity penalty are weighted by their respective scaling factors (i.e., SPF and CSM). “Cost” is measured as number of actions selected; “Species penalty” as the number of sites where each species does not have a benefit of 1; and “Connectivity penalty” as the inverse of the squared distance (1/km2) between pairs of sites, where one of the sites is not in the solution. (TIF) [file pone.0128027.s004.tif]

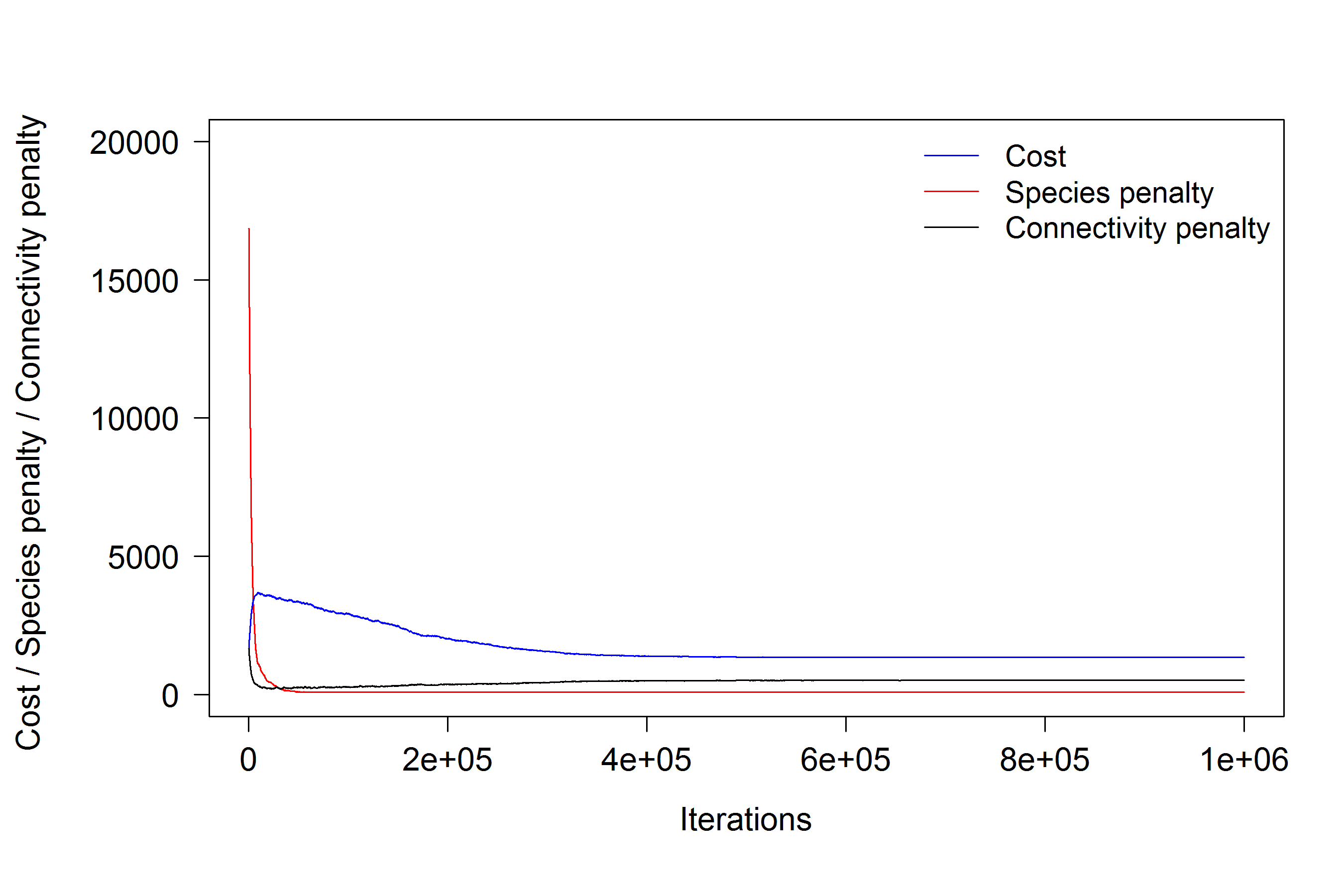

Supplement: S3 Fig — The values of Species and Connectivity penalty are weighted by their respective scaling factors (i.e., SPF and CSM). “Cost” is measured as number of actions selected; “Species penalty” as the number of sites where each species does not have a benefit of 1; and “Connectivity penalty” as the inverse of the squared distance (1/km2) between pairs of sites, where one of the sites is not in the solution. (TIF) [file pone.0128027.s005.tif]

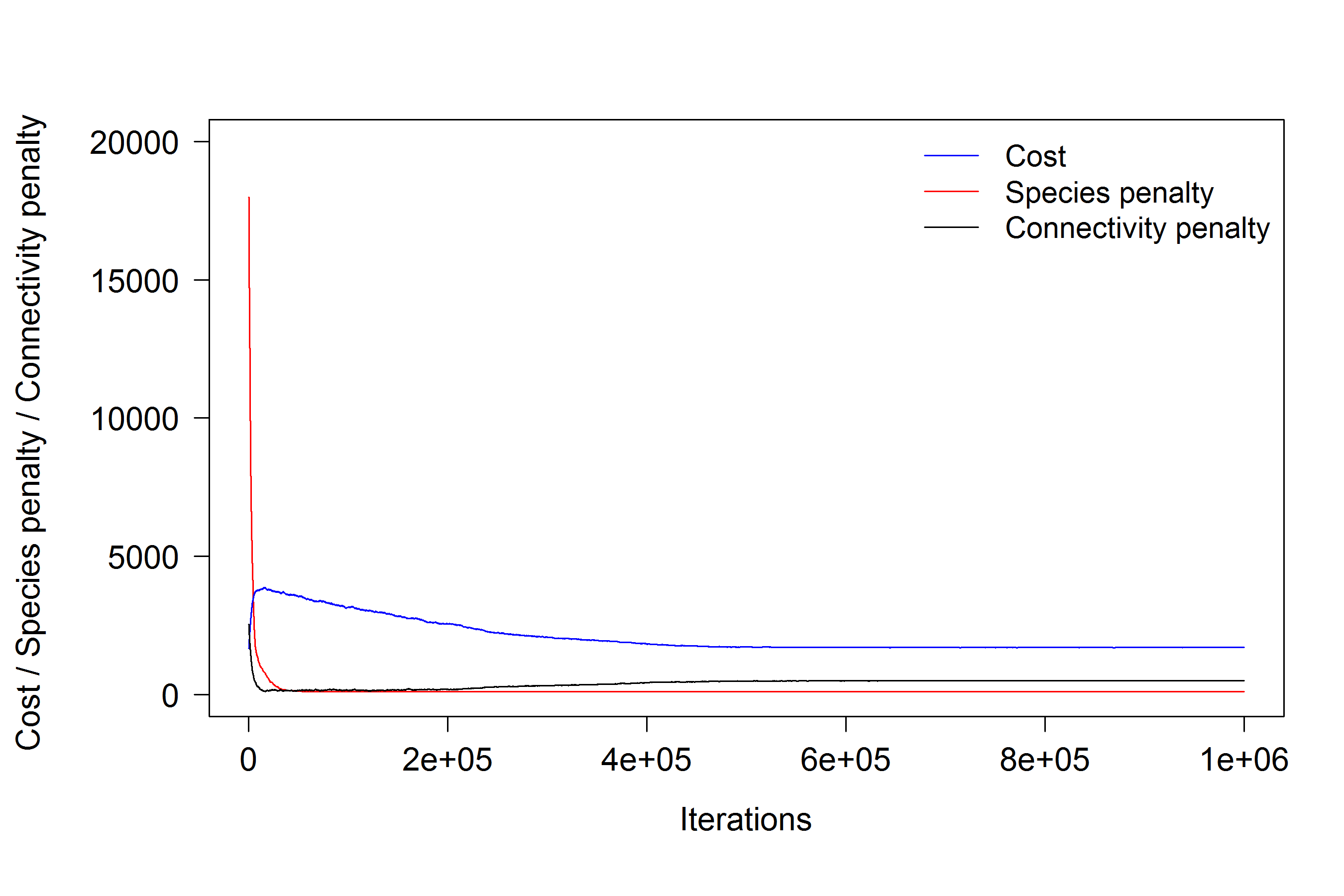

Supplement: S4 Fig — The values of Species and Connectivity penalty are weighted by their respective scaling factors (i.e., SPF and CSM). “Cost” is measured as number of actions selected; “Species penalty” as the number of sites where each species does not have a benefit of 1; and “Connectivity penalty” as the inverse of the squared distance (1/km2) between pairs of sites, where one of the sites is not in the solution. (TIF) [file pone.0128027.s006.tif]

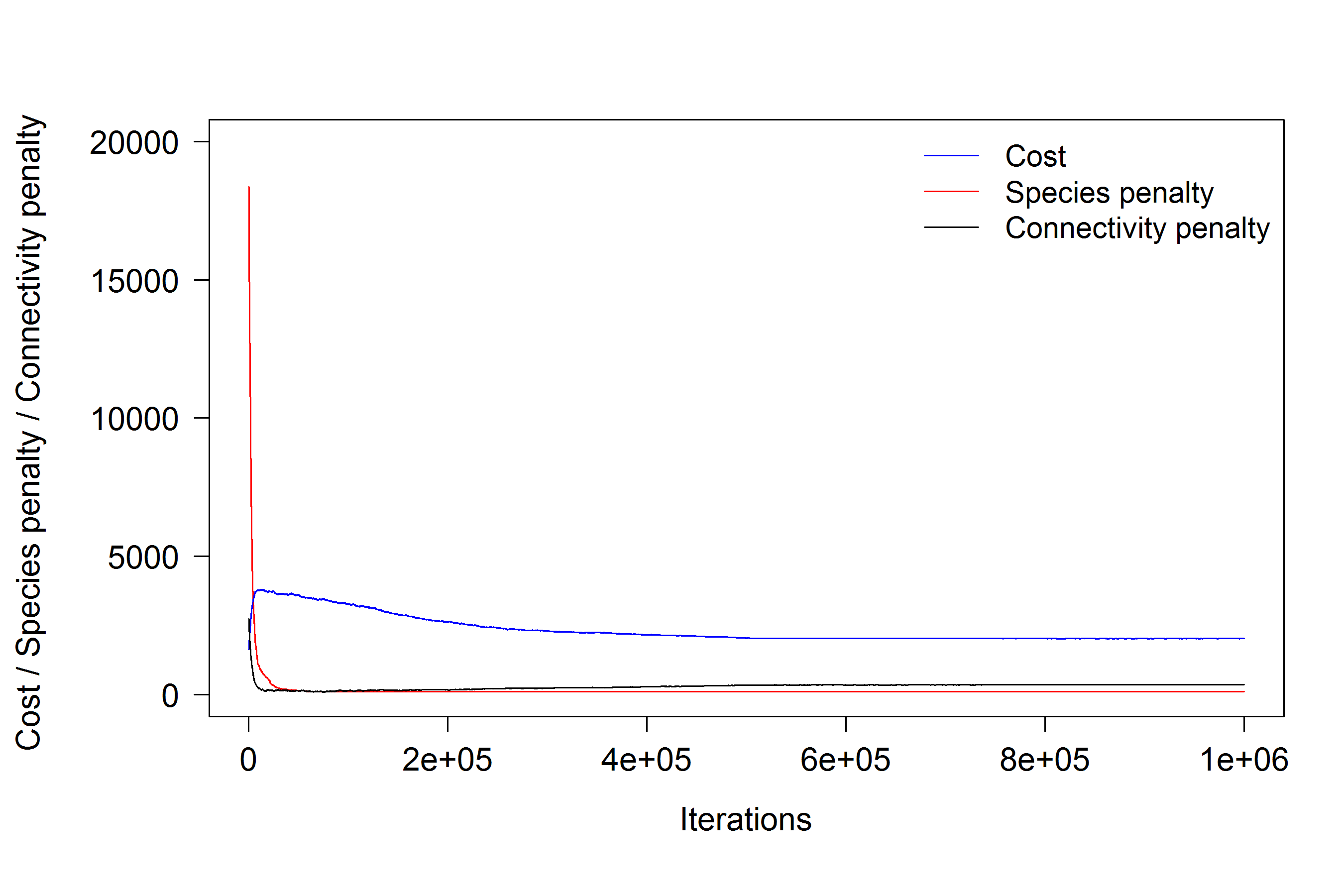

Supplement: S5 Fig — The values of Species and Connectivity penalty are weighted by their respective scaling factors (i.e., SPF and CSM). “Cost” is measured as number of actions selected; “Species penalty” as the number of sites where each species does not have a benefit of 1; and “Connectivity penalty” as the inverse of the squared distance (1/km2) between pairs of sites, where one of the sites is not in the solution. (TIF) [file pone.0128027.s007.tif]

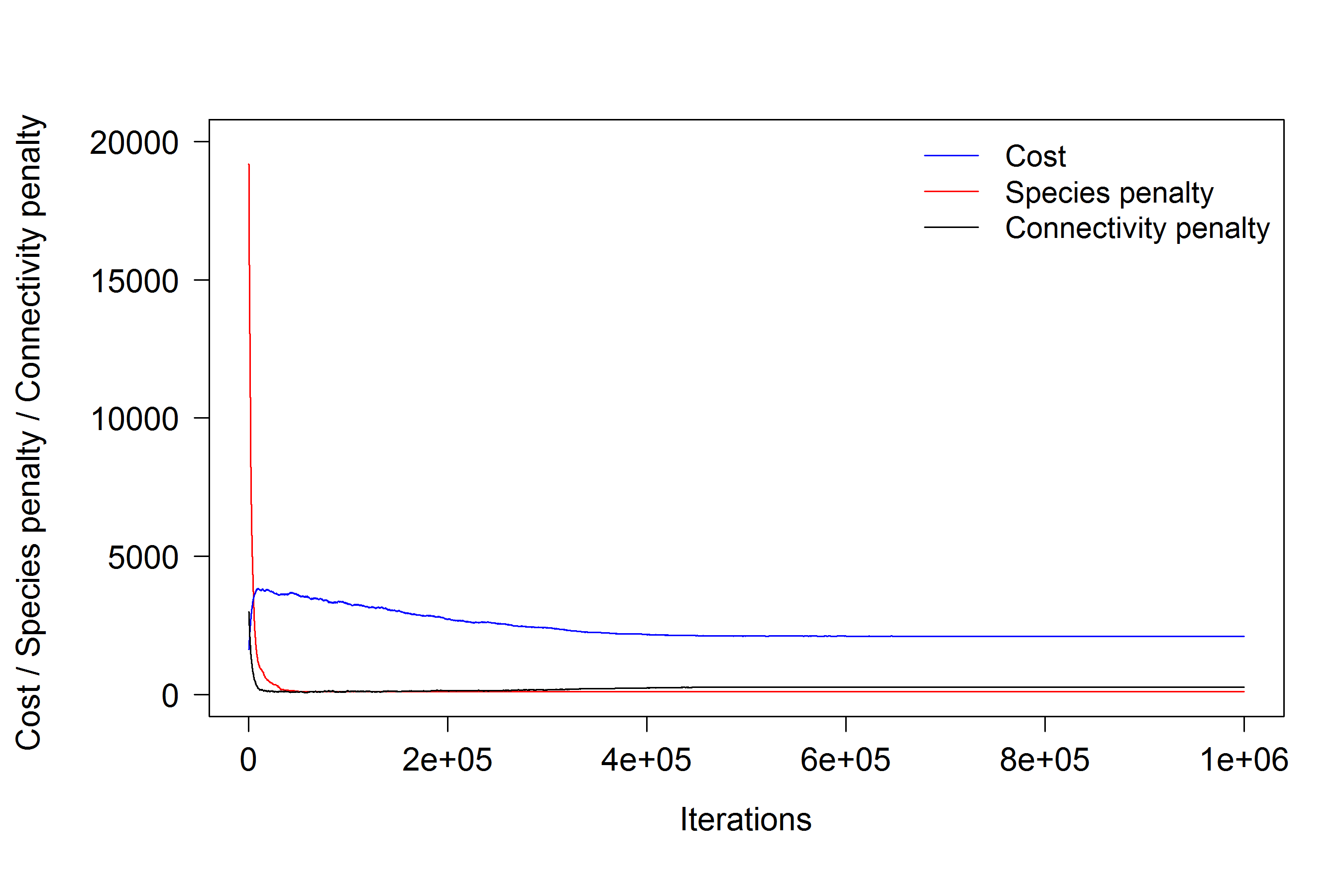

Supplement: S6 Fig — The values of Species and Connectivity penalty are weighted by their respective scaling factors (i.e., SPF and CSM). “Cost” is measured as number of actions selected; “Species penalty” as the number of sites where each species does not have a benefit of 1; and “Connectivity penalty” as the inverse of the squared distance (1/km2) between pairs of sites, where one of the sites is not in the solution. (TIF) [file pone.0128027.s008.tif]

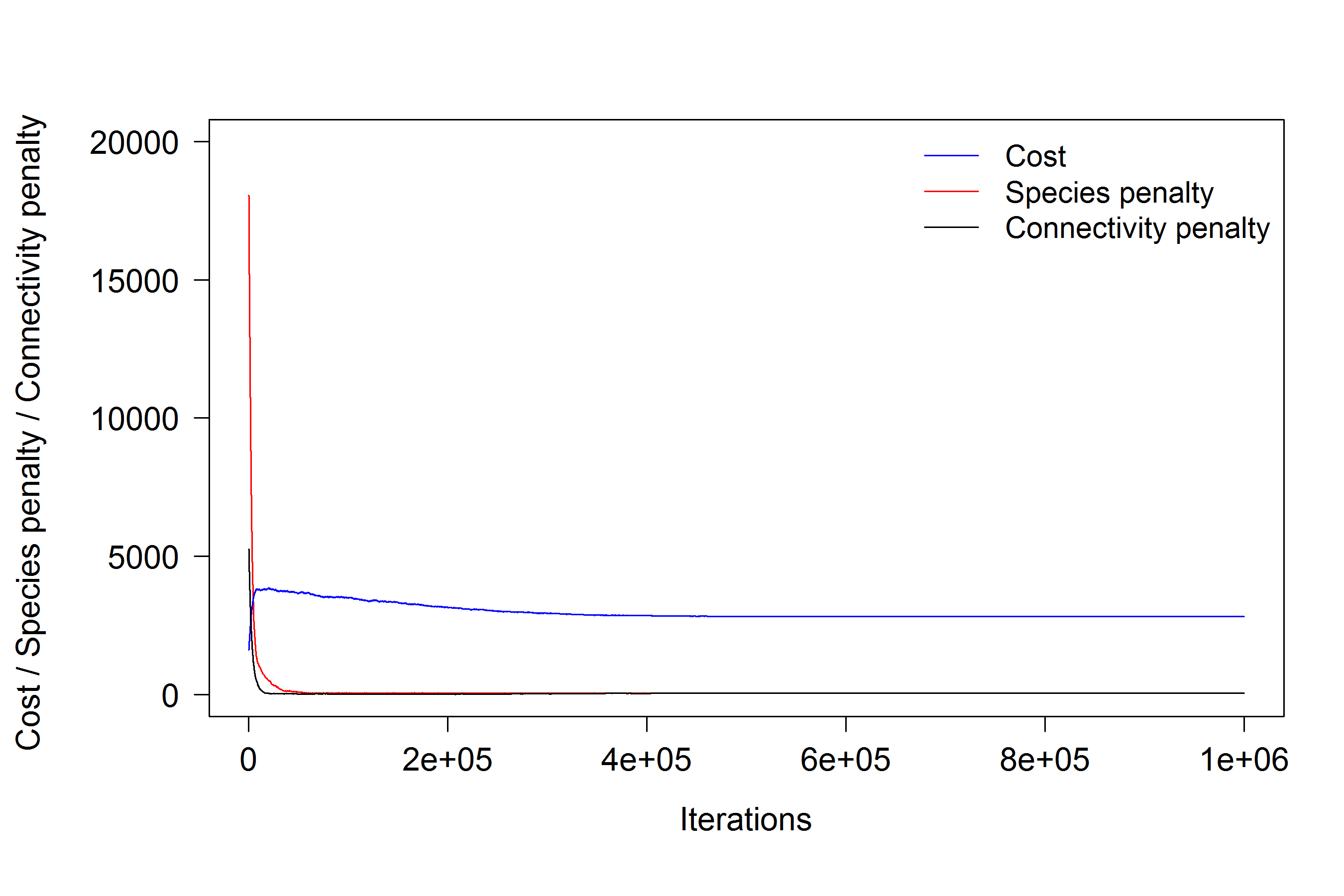

Supplement: S7 Fig — The values of Species and Connectivity penalty are weighted by their respective scaling factors (i.e., SPF and CSM). “Cost” is measured as number of actions selected; “Species penalty” as the number of sites where each species does not have a benefit of 1; and “Connectivity penalty” as the inverse of the squared distance (1/km2) between pairs of sites, where one of the sites is not in the solution. (TIF) [file pone.0128027.s009.tif]

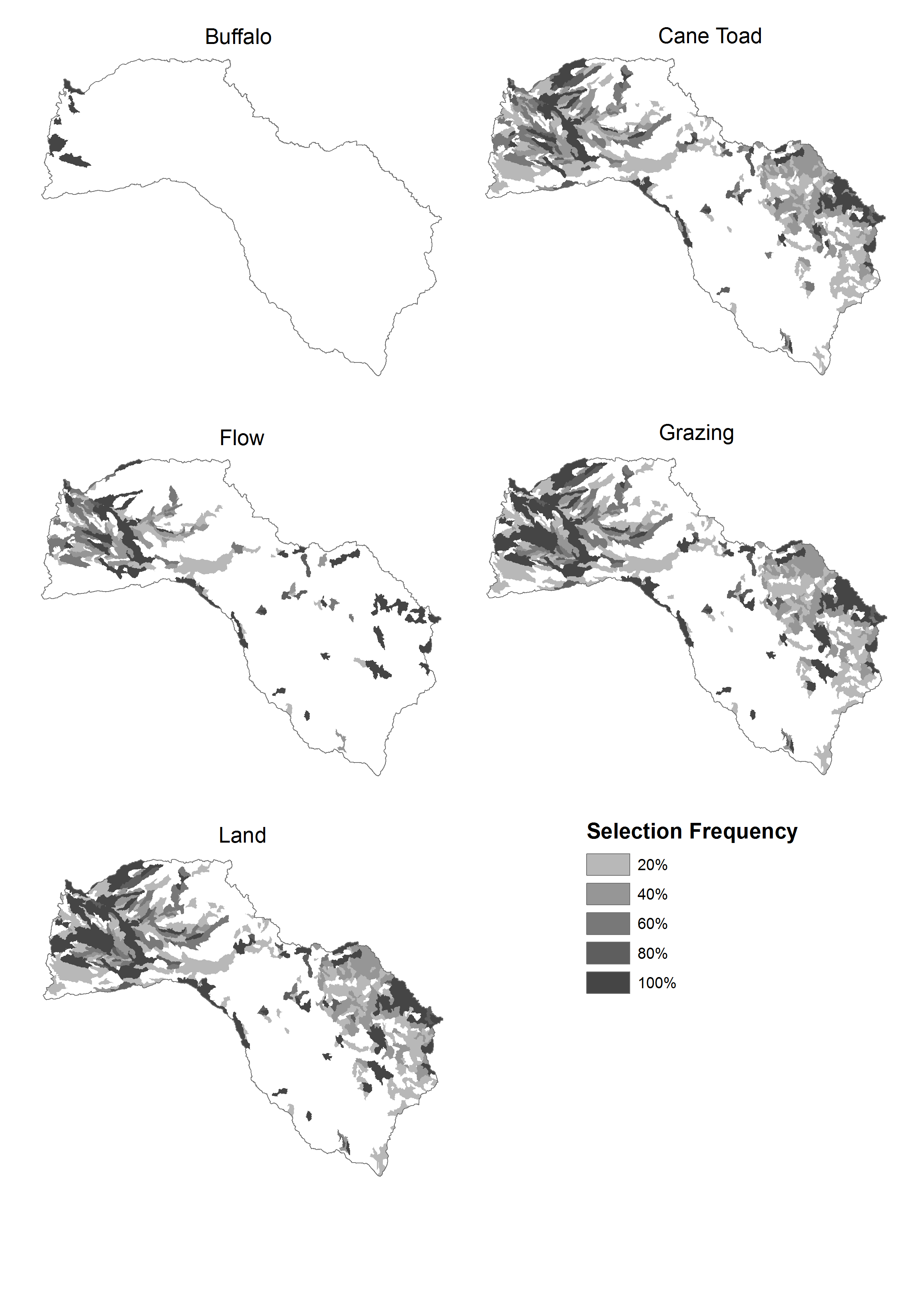

Supplement: S8 Fig — Selection frequency is calculated as the number of times each action is selected across 100 replicates. Actions are: buffalo control (“Buffalo”), cane toad control (“Cane Toad”), river flow-regime restoration (“Flow”), grazing management (“Grazing”) and land acquisition (“Land”). (TIF) [file pone.0128027.s010.tif]

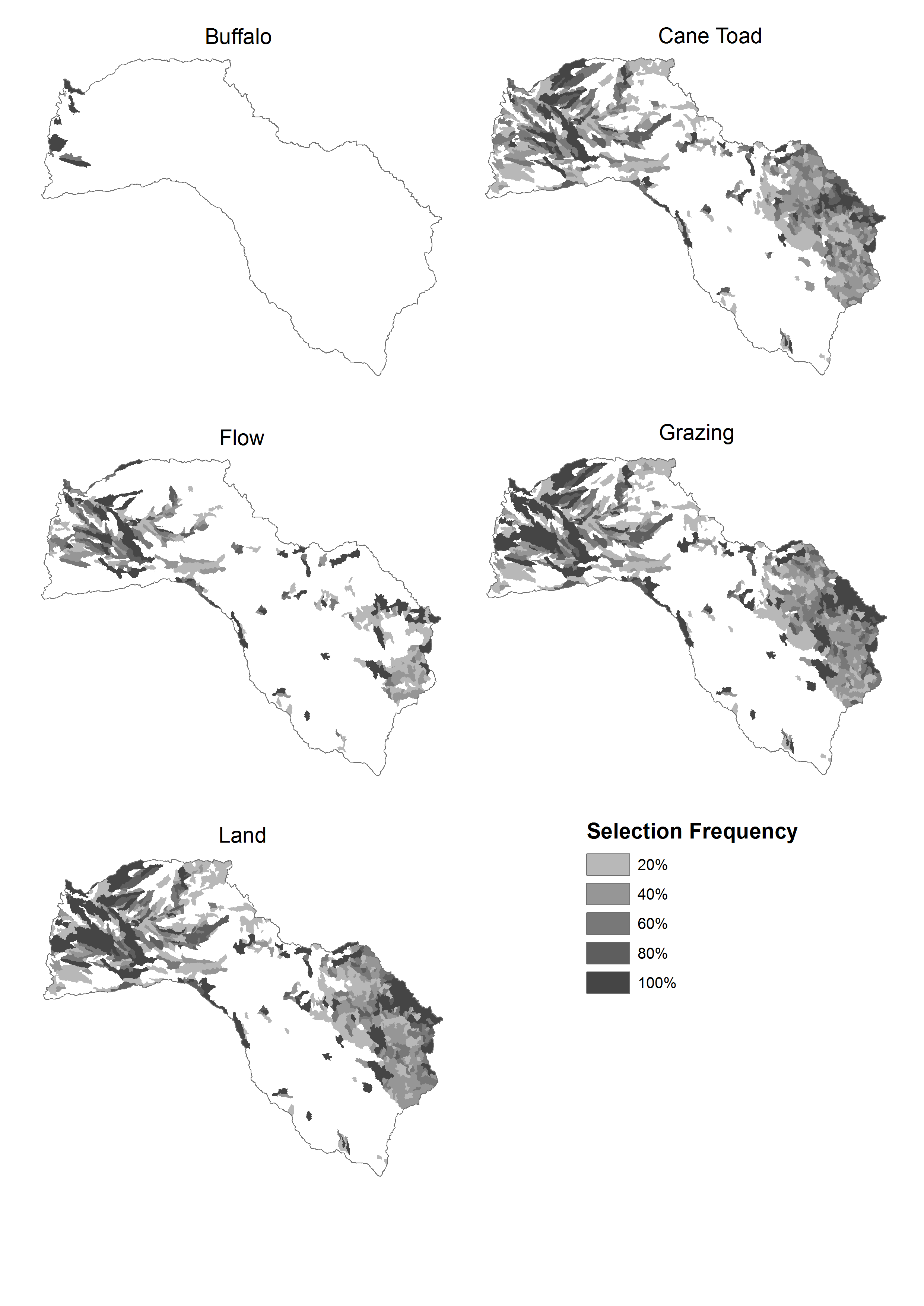

Supplement: S9 Fig — Selection frequency is calculated as the number of times each action is selected across 100 replicates. Actions are: buffalo control (“Buffalo”), cane toad control (“Cane Toad”), river flow-regime restoration (“Flow”), grazing management (“Grazing”) and land acquisition (“Land”). (TIF) [file pone.0128027.s011.tif]

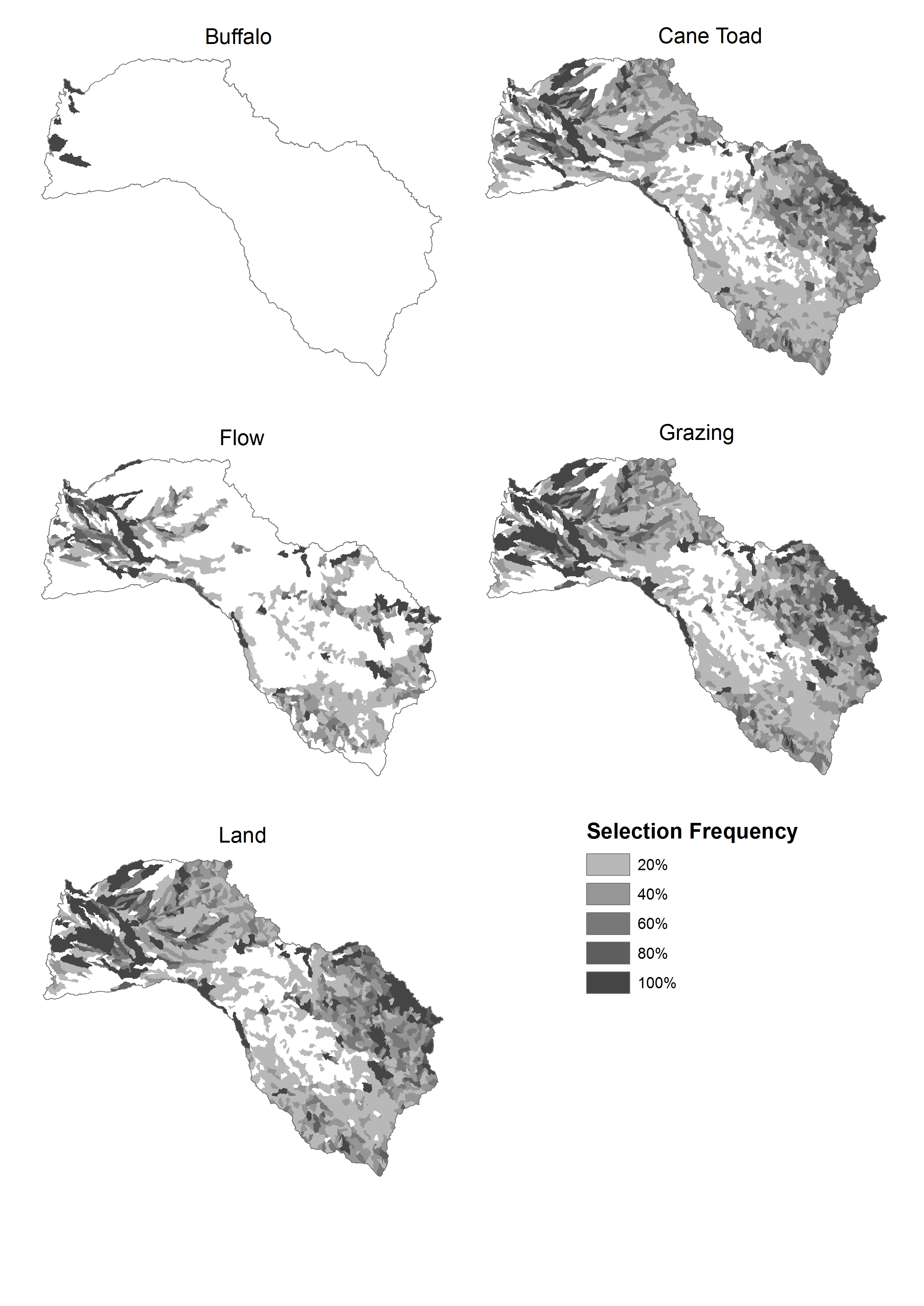

Supplement: S10 Fig — Selection frequency is calculated as the number of times each action is selected across 100 replicates. Actions are: buffalo control (“Buffalo”), cane toad control (“Cane Toad”), river flow-regime restoration (“Flow”), grazing management (“Grazing”) and land acquisition (“Land”). (TIF) [file pone.0128027.s012.tif]

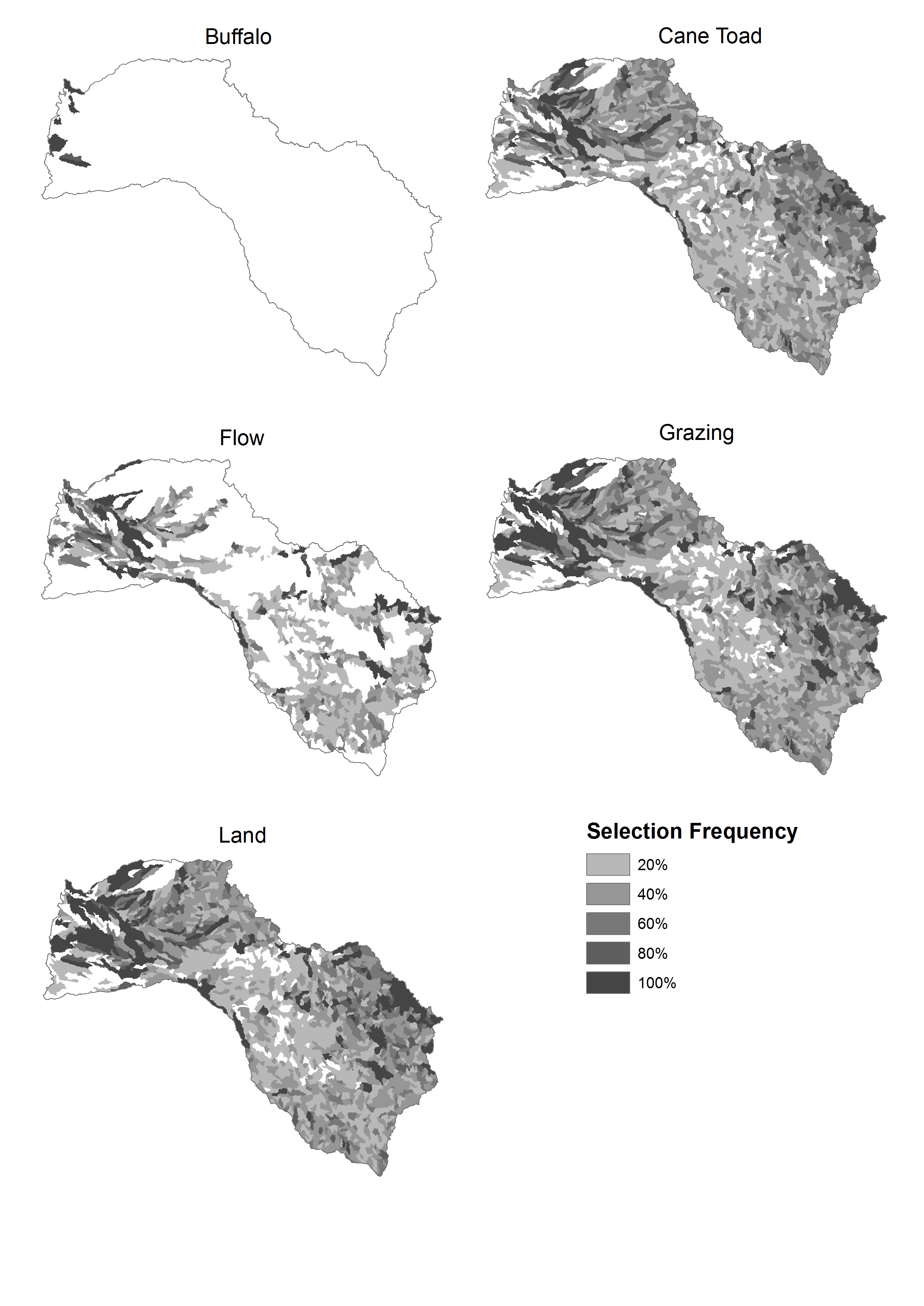

Supplement: S11 Fig — Selection frequency is calculated as the number of times each action is selected across 100 replicates. Actions are: buffalo control (“Buffalo”), cane toad control (“Cane Toad”), river flow-regime restoration (“Flow”), grazing management (“Grazing”) and land acquisition (“Land”). (TIF) [file pone.0128027.s013.tif]

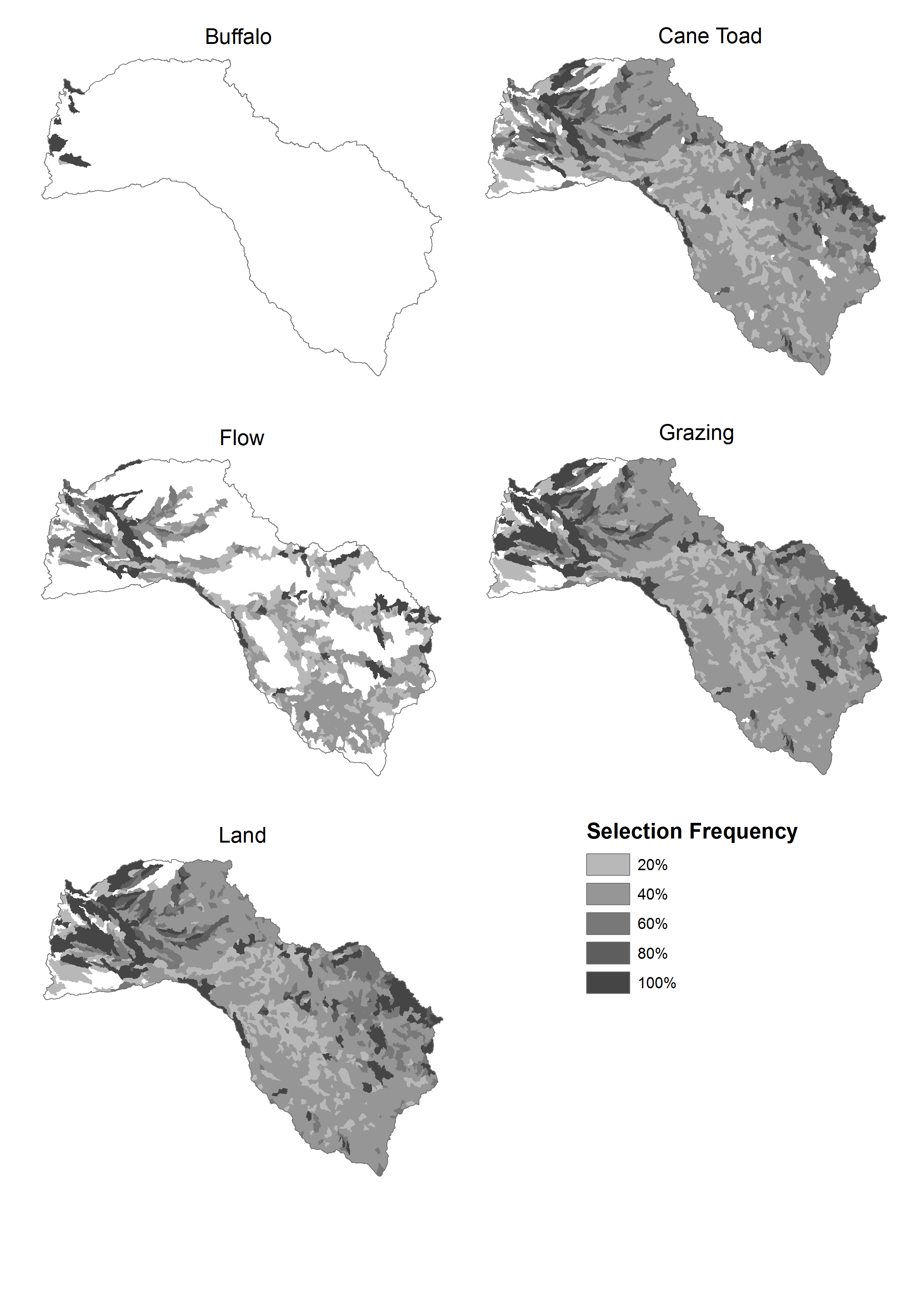

Supplement: S12 Fig — Selection frequency is calculated as the number of times each action is selected across 100 replicates. Actions are: buffalo control (“Buffalo”), cane toad control (“Cane Toad”), river flow-regime restoration (“Flow”), grazing management (“Grazing”) and land acquisition (“Land”). (TIF) [file pone.0128027.s014.tif]

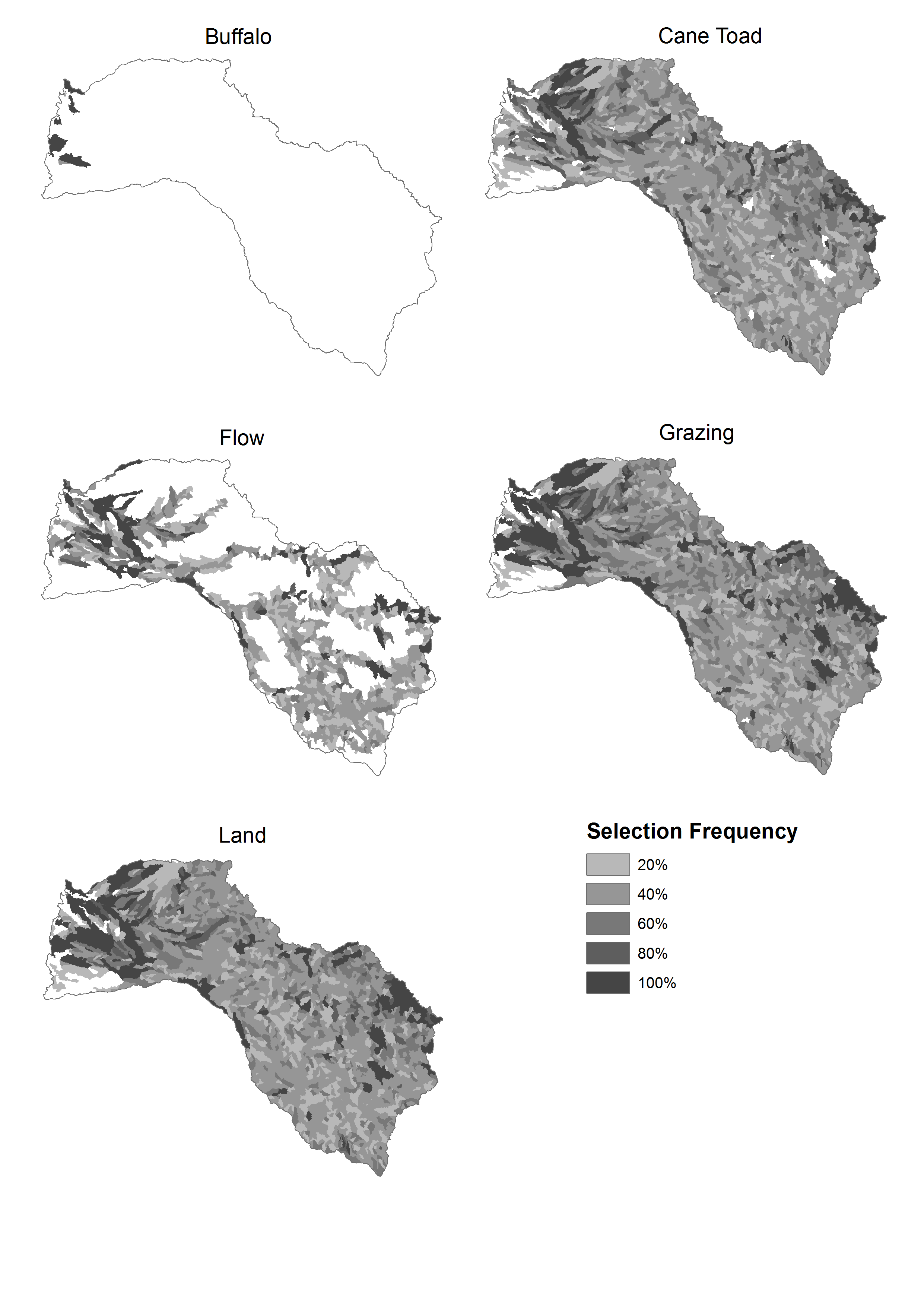

Supplement: S13 Fig — Selection frequency is calculated as the number of times each action is selected across 100 replicates. Actions are: buffalo control (“Buffalo”), cane toad control (“Cane Toad”), river flow-regime restoration (“Flow”), grazing management (“Grazing”) and land acquisition (“Land”). (TIF) [file pone.0128027.s015.tif]
